# Injectable magnetic montmorillonite colloidal gel for the postoperative treatment of hepatocellular carcinoma

**DOI:** 10.1186/s12951-022-01559-7

**Published:** 2022-08-19

**Authors:** Sheng Chen, Yonghong Song, Xu Yan, Liang Dong, Yunjun Xu, Shouhu Xuan, Quan Shu, Baoqiang Cao, Jinlong Hu, Hanye Xing, Wenshu Wu, Zhengbao Zha, Yang Lu

**Affiliations:** 1 Anhui Key Laboratory of Advanced Catalytic Materials and Reaction Engineering, School of Chemistry and Chemical Engineering, School of Food and Biological Engineering, Hefei University of Technology, Hefei, 230009 China; 2 Division of Nanomaterials & Chemistry, Department of Modern Mechanics, Department of Radiology, Hefei National Laboratory for Physical Sciences at the Microscale, The First Affiliated Hospital of University of Science and Technology of China, University of Science and Technology of China, Hefei, 230026 China; 3 Department of General Surgery, Department of Ultrasonics, Department of Interventional Radiology, Anhui No. 2 Provincial People’s Hospital, Hefei, 230041 Anhui China; 4 Chinese Academy of Sciences, Institute of Basic Medicine and Cancer (IBMC), The Cancer Hospital of the University of Chinese Academy of Sciences (Zhejiang Cancer Hospital), Hangzhou, 310022 Zhejiang China

**Keywords:** Colloidal gel, Magnetic hyperthermia, Hepatocellular carcinoma, Postoperative therapy, Hemostasis

## Abstract

Bioactive materials have been extensively developed for the adjuvant therapy of cancer. However, few materials can meet the requirements for the postoperative resection of hepatocellular carcinoma (HCC) due to massive bleeding and high recurrence. In particular, combination therapy for HCC has been highly recommended in clinical practice, including surgical resection, interventional therapy, ablation therapy and chemotherapy. Herein, an injectable magnetic colloidal gel (MCG) was developed by controllable electrostatic attraction between clinically available magnetic montmorillonites and amphoteric gelatin nanoparticles. The optimized MCG exhibited an effective magnetic heating effect, remarkable rheological properties, and high gel network stability, realizing the synergistic treatment of postoperative HCC by stimuli-responsive drug delivery, hemostasis and magnetic hyperthermia. Furthermore, a minimal invasive MCG-induced interventional magnetic hyperthermia therapy (MHT) under ultrasound guidance was realized on hepatic tumor rabbits, providing an alternative therapeutics to treat the postoperative recurrence. Overall, MCG is a clinically available injectable formulation for adjuvant therapy after HCC surgical resection.

**Supplementary Information:**

The online version contains supplementary material available at 10.1186/s12951-022-01559-7.

## Introduction

Hepatocellular carcinoma (HCC) is one of the most common malignant tumors in the world, the incidence rate ranks sixth among all cancers, and the fatality rate ranks third [1]. Surgical resection is the main treatment for HCC, but the recurrence rate of liver cancer within 5 years is as high as 50–70% owing to the residual microscopic tumor cells and the lack of effective adjuvant treatments [2]. Systemic chemotherapy is widely used to prevent tumor recurrence in postoperative patients. Unfortunately, systemic chemotherapy leads inevitably to systemic toxicities that exacerbate the damage to the human body [3–5]. Local drug delivery implants have been used to achieve decreased systemic side effects and more efficient therapeutics [6–13]. Another challenge facing caregivers is uncontrolled intraoperative blood loss during HCC surgical resection. According to previous reports, hemorrhage not only causes poor patient prognosis but also presents great challenges for the in situ administration of adjuvant treatments [14–16]. Pioneering topical hemostatic implants, including sponges and gauze, loaded with antitumor drugs are widely reported to stop bleeding and achieve local chemotherapy [9, 17], but these implants have poor fluidity and are thus unable to match the irregular shape of the HCC postoperative cavity, leading to uneven drug distribution and inhibiting the performance of the implant. A promising alternative to the postoperative treatment of cancer is to use an injectable supramolecular hydrogel with thermosensitive fluidity. By virtue of its remarkable fluidity, this hydrogel achieves covers of all residual cancerous cells and prevents the recurrence of cancer [18–20]. Nevertheless, the application of such chemically cross-linked hydrogels has been restricted by the potential toxicity of the residual functional groups, organic cross-linkers and catalysts, so the biocompatibility of hydrogel needs to be further improved [21]. Therefore, there is a strong clinical need for the design of an injectable and biocompatible hydrogel with the desired fluidity and hemostatic abilities to achieve effective postoperative adjuvant treatments of HCC [22].

Colloidal gels with self-assembled particulate networks have become desirable candidates for local drug delivery implants [23, 24]. Compared with conventional monolithic hydrogels, colloidal gels are composed solely of colloidal particles, and better control over their physicochemical properties can be achieved by fine tuning the characteristics of the building blocks [25, 26]. Attractively, by taking advantage of noncovalent bonds, self-healing colloidal gels can be constructed with remarkable injectability and high sensitivity to various stimuli, which are beneficial properties for local drug release at the tumor surgical margin [18]. Therapeutic polydopamine nanoparticles have been employed by our group as electronegative building blocks to construct colloidal gels, which achieved the near-infrared light (NIR)-triggered chemothermal cotherapy of tumors [27]. However, NIR has difficulty penetrating deep tissue, therefore, this colloidal gel was hard to provide noninvasive, long-term, effective treatment for deep tumors such as HCC. Alternating magnetic field (AMF) with limitless tissue depth has been used for magnetic nanomaterial-induced magnetic hyperthermia therapy (MHT), which was approved by European regulatory agencies and has achieved great clinical success for deep-seated cancers such as malignant glioma, prostate cancer and brain cancer [28–30]. Furthermore, remote manipulations and controllable drug release of magnetic hydrogels under AMF have been achieved [31–33].

**Scheme 1:**
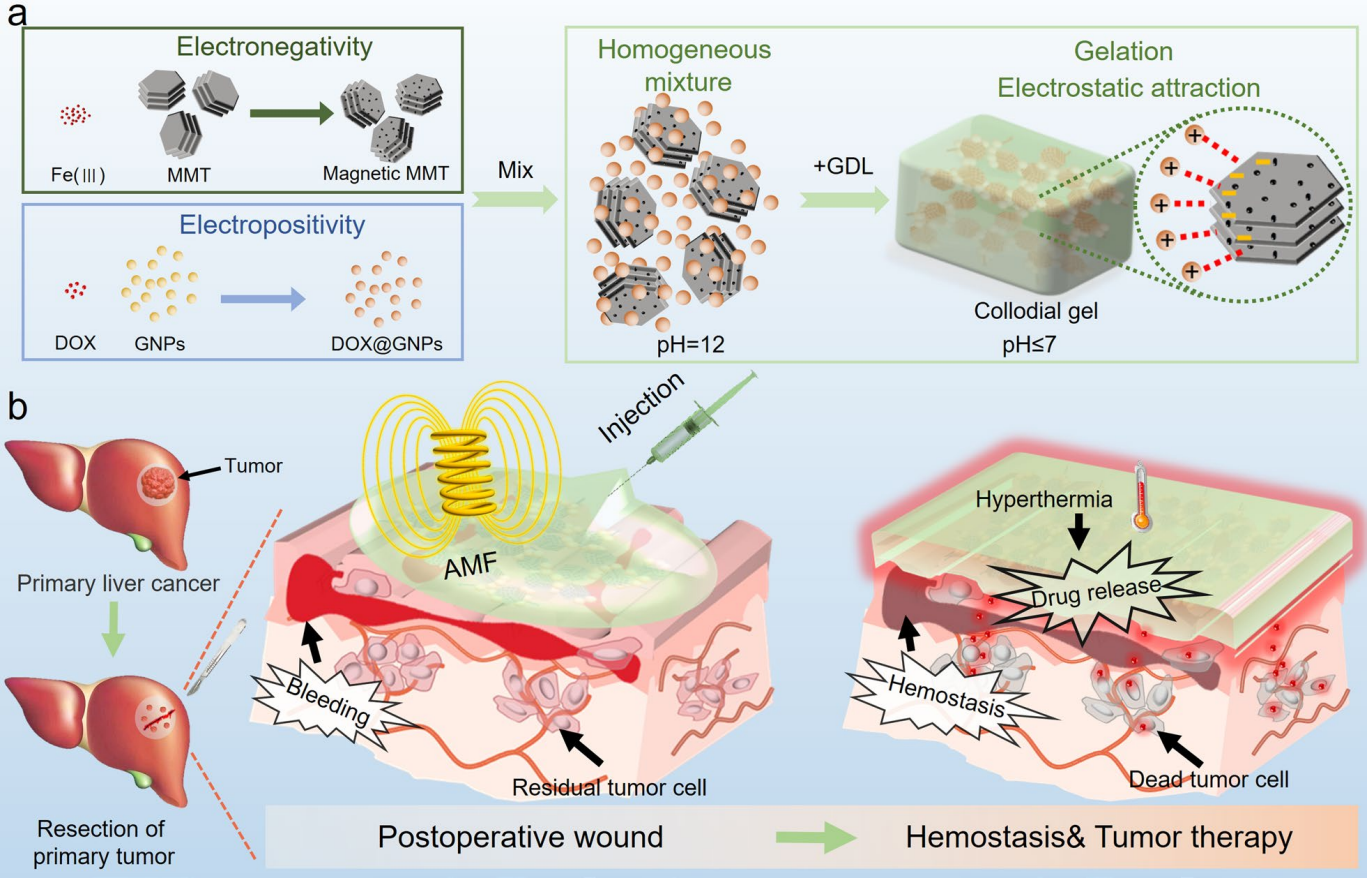
Scheme of the formation and function of the composite colloidal gel. **a** The forming process of MCG_*DOX*_ from two building blocks (magnetic MMT and DOX@GNPs). **b** Scheme of the synergistic mechanism of injectable MCG_*DOX*_ on hemostasis, magnetic hyperthermia, and controllable drug release in the HCC regions after surgical resection

Herein, we developed injectable magnetic colloidal gels (MCGs) assembled from magnetic montmorillonites (MMTs) and gelatin nanoparticles (GNPs) to satisfy the needs of complicated HCC postoperative environments, such as counteracting postoperative bleeding and tumor recurrence. GNPs were used as positively charged building blocks due to their biocompatibility and good drug loading capacity [27]. MMT, a natural phyllosilicate, has been used as an oral agent for more than 30 years in the clinic [34]. Moreover, MMT is an effective hemostatic agent because its negative charge stimulates blood coagulation [35]. As shown in Scheme 1a, magnetic MMT was synthesized by a facile in situ method and served as the magnetic negatively charged building block. The colloidal particles were first mixed in alkaline conditions (pH = 12) to maintain electrostatic repulsion between particles and achieve homogeneous mixing. D-(+)-gluconic acid δ-lactone (GDL) was introduced as an acidifier that induces the charge reversal of GNPs. Finally, the MCG was obtained by applying an electrostatic force. Composites of organic/inorganic building blocks were useful for improving the mechanical properties and three-dimensional network stability of gels. Therefore, introducing the inorganic building block of magnetic MMT into the colloidal gel can lead to the fabrication of MCG with (1) strong hemostatic ability, (2) intelligent responsivity under a magnetic field, and (3) a gel network with excellent stability, enriching the feasibility of the MCG for broad biomedical applications. The heating response under AMF enables MCG_*DOX*_ with fluidity to fill the irregular surgical margin and achieve hemostasis, further combining chemotherapy with magnetic hyperthermia to kill residual cancer cells after HCC surgery (Scheme 1b). The recurrence time of HCC would be effectively delayed after these well-designed postoperative adjuvant therapy strategies implemented. When the secondary surgical resection was not recommended, percutaneous local ablation therapy was considered as a clinically curative treatment for postoperative recurrence [36–40]. MCG provided an alternative minimal invasive treatment and achieved successful interventional MHT under ultrasound guidance on VX2 tumor rabbits, which effectively prevented the postoperative recurrence. Collectively, MCG with a sensitive response to AMF is a reasonable candidate for achieving minimal invasive, long-term postoperative adjuvant therapy of deep hepatic tumors.

## Materials and methods

### Materials

Gelatin A (from porcine skin, 300 Bloom), Iron (III) acetylacetonate (≥ 99.9%) and Poly(sodium 4-styrenesulfonate) (PSS, M_w_ ~ 70,000) were purchased from Sigma-Aldrich Co. Ltd. D-(+)-gluconic acid δ-lactone (GDL; ≥ 99.0%), Glutaraldehyde solution (25%), Doxorubicinol (97%), Tetraethylene glycol (99%) and Glycine (> 99%) were obtained from Aladdin Reagent Co. Ltd. Acetone (> 99%) was purchased from Sinopharm Chemical Reagent Co. Ltd. Montmorillonite was obtained from Zhejiang Sanding Technology Co. Ltd. All chemicals were used without any purification unless otherwise specified.

### Characterizations

SEM images were obtained using a scanning electron microscope (Zeiss Supra 40, Germany). The particle size was evaluated by a dynamic light scattering instrument (Malvern Zetasizer, Nano-ZS90, UK). XRD pattern was obtained using a Philips X’ Pert Pro SUPER instrument (Netherland). Super Rheological characterizations were performed by a rotational rheometer (Anton Paar, MCR 302, Austria). The injection force of the MCG was measured using a mechanical testing system (Instron 5965, USA). The MHT was performed using a high frequency (312 kHz) heating equipment (Shuangping SPG, China).

### Synthesis of magnetic MMT particles

Firstly, the dispersing liquid with 100 mg MMT and PSS (10 mg/mL) was shaken by vortex for 3 min and stood for 30 min. Then the mixture was centrifugally washed (3000 rpm, 10 min) three times by deionized water and freeze-dried, the obtained product was MMT-PSS. The above MMT-PSS and different mass (50, 100, 200, 400 mg) iron acetylacetonate were added to 50 mL of triethylene glycol, and then the mixture was put into a three-necked reaction flask. The above mixture was heated to 300 °C at a rate of 3 °C/min and kept at 300 °C for 30 min. The obtained solution was centrifuged at 3000 rpm (10 min) and washed with anhydrous ethanol three times.

### Preparation of composite MCG

According to the previous report [24], GNPs were prepared by a two-step desolvation method. A certain amount of GNPs and magnetic MMT were mixed by vortex at alkaline condition (pH ≈ 12), and 80 mM GDL powder was added to induce gelation. MCGs with different solids content (5%, 10%, 15% w/v) were synthesized by the above method, and the mass ratio of magnetic MMT to GNPs was 1:1. Specifically, three MCGs (10% solids content) with different feed ratios were prepared. MMT and GNPs were mixed in the ratio 1:3, 1:1, and 3:1 by mass. Pure MMT without magnetic nanoparticles was also used to prepare composite colloidal gels with a mass/volume fraction of 10%.

In addition, DOX@GNPs were first prepared to fabricate DOX-loaded MCGs. Herein, DOX was loaded through adsorption by GNPs. Specifically, 100 mg GNPs power was added into pre-prepared DOX solution (1 mg/mL, 1 mL), until the GNPs were fully swelling. DOX was adsorbed by swelled GNPs and the DOX@GNPs was obtained.

### Rheological characterizations

The rheological properties of MCGs were tested by a rotational rheometer with a rotating plate and a gap distance of 1000 μm. The sol-gel process was determined by the oscillation time to sweep measurement with a frequency of 1 Hz and a strain of 0.5%. Frequency sweep measurement (0.1–100 Hz) at the fixed strain of 1%. Strain scanning measurements were performed at a constant frequency of 1 Hz with a strain range of 1–500%. Self-healing properties of the colloidal gels were performed by multiple continuous strain and time scan (5 min) cycles.

### In vitro and in vivo toxicity evaluation

The CCK-8 method was used to determine the safety of MCG in vitro. NIH-3T3 cells (Guangzhou Cellcook Biotechnology Co. Ltd., China) were cultured in Dulbecco’s modified eagle medium (DMEN) at 37 °C with 5% CO_2_ concentration. To evaluate the cellular safety of the material, NIH-3T3 cells were seeded into 96-well plates. Then, the cells were co-inoculated with magnetic MMT, GNPs and MCG for 24 h. Cell activity was detected by a standard CCK-8 method. 10 µL CCK-8 solution (Beyotime Biotechnology Co. Ltd. Shanghai, China) was added to each well, the cells were further incubated in the cell incubator for 2 h, then the absorbance at 450 nm of each well was measured on the microplate reader (Themo, Multiskan FC, USA) to calculate the cell survival rate.

To evaluate the in vivo toxicity, MCG (100 µL) was subcutaneously implanted into healthy ICR mice (6–8 weeks old, Anhui Medical University), and mice injected subcutaneously with PBS were considered as the control group. After 14 days, skins around implants and blood samples were collected. The typical Masson’s trichrome and Hematoxylin & Eosin (H&E) tissue stainings were applied to evaluate the pathological changes of MCG and PBS implantation sites. The obtained blood samples were used for the analysis of main serum parameters.

### In vitro coagulation test

According to the method of the previous report [41], the hemostatic effect of MCG was evaluated in vitro. As a control group, 50 µL of blood was dropped directly into the petri dish. At the same time, gelfoam (Jiangxi Xiangen Medical Technology Development Co., Ltd. China) and MCG (100 µL) were placed into two petri dishes, respectively. Then, 50 µL of blood was dropped onto the samples. After 5 min, 10 mL distilled water was added slowly without stirring the coagulated blood. Each sample was slightly shaken to dissolve free red blood cell. The hemoglobin absorbance of each sample was measured at 542 nm by an ultraviolet spectrophotometer. The blood clotting index (BCI) was calculated according to the equation: BCI = A_M_/A_W_×100% (A_M_: the absorbency of blood mixed with various materials at 542 nm. A_W_: the absorbency of whole blood in distilled water at 542 nm).

### In vivo evaluation of the hemostatic performance

The hemostatic performance of MCG was evaluated in rat hepatic hemorrhage model. The livers of three SD rats (male, 180–200 g, n = 3) were exposed through an abdominal incision and scratched to bleeding with a scalpel. Immediately, MCG and Gelfoam were administered at the bleeding site, respectively. The third rat has not received any treatment at the bleeding site. The loss blood of different treatment groups was collected and analyzed quantitatively. In addition, the rabbit orthotopic transplanted HCC model was established by implanting the VX2 tumor tissue into the liver of New Zealand rabbit (2–3 kg, Anhui Medical University). Two weeks after implant, the tumor tissues were surgically removed. Subsequently, MCG was injected onto the surgical site to effectively stop bleeding, and then the liver surgical site was exposed to AMF for 10 min for further MHT.

### Evaluation of the effect of preventing tumor recurrence

To establish HepG2 tumor-bearing mice, Balb/c female mice (6–8 weeks, Anhui Medical University) received a subcutaneous injection of HepG2 cells (1 × 10^6^, 200 µL PBS, iCell Bioscience Inc, Shanghai, China). Ten days after HepG2 cells inoculation, the tumor-bearing mice were randomly divided into 5 groups (5 mice per group). The tumor tissues were surgically removed, and then five groups of mice received treatment of (1) MCG (100 µL), (2) MCG_*DOX*_ (100 µL), (3) MCG (100 µL) + AMF, (4) MCG_*DOX*_ (100 µL) + AMF, and (5) only underwent surgical resection. Subsequently, the mice were exposed to AMF (H = 30 kA/m, f = 312 kHz) for 15 min. The temperature of the tumor site was monitored by an infrared thermography camera (Fluke, Ti400). The body weight and tumor volume of mice were measured every 2 days, and the tumor recurrence of mice in each group was recorded every two days for a total of 14 days.

$$V=\frac{L\times {W}^{2}}{2}$$

Individual tumor volume (V) was calculated according to the formula, where length (L) was the maximum diameter of the tumor, and width (W) was the shortest diameter perpendicular to the length of the tumor.

### Interventional thermotherapy of HCC

The New Zealand rabbit with VX2 liver cancer was used to evaluate the feasibility of MCG in the treatment of liver cancer by interventional puncture injection and magnetic hyperthermia (n = 3). MCG was minimally injected into the site of a tumor by percutaneous puncture under ultrasound guidance (Hitachi Aloka Arietta 70). Subsequently, the rabbits were exposed to AMF (H = 30 kA/m, f = 312 kHz) for 15 min. After MHT, tissues in tumor sites were removed and analyzed by H&E.

### Statistical analysis

All results were expressed as mean ± SD. All the error bars were standard deviations. Mean, standard deviation, and p values were calculated in Microsoft Excel. The experimental data were analyzed by unpaired student’s T test. *p ≤ 0.05, **p ≤ 0.01, ***p ≤ 0.001.

## Results and discussion

### Preparation of MCG

**Fig. 1:**
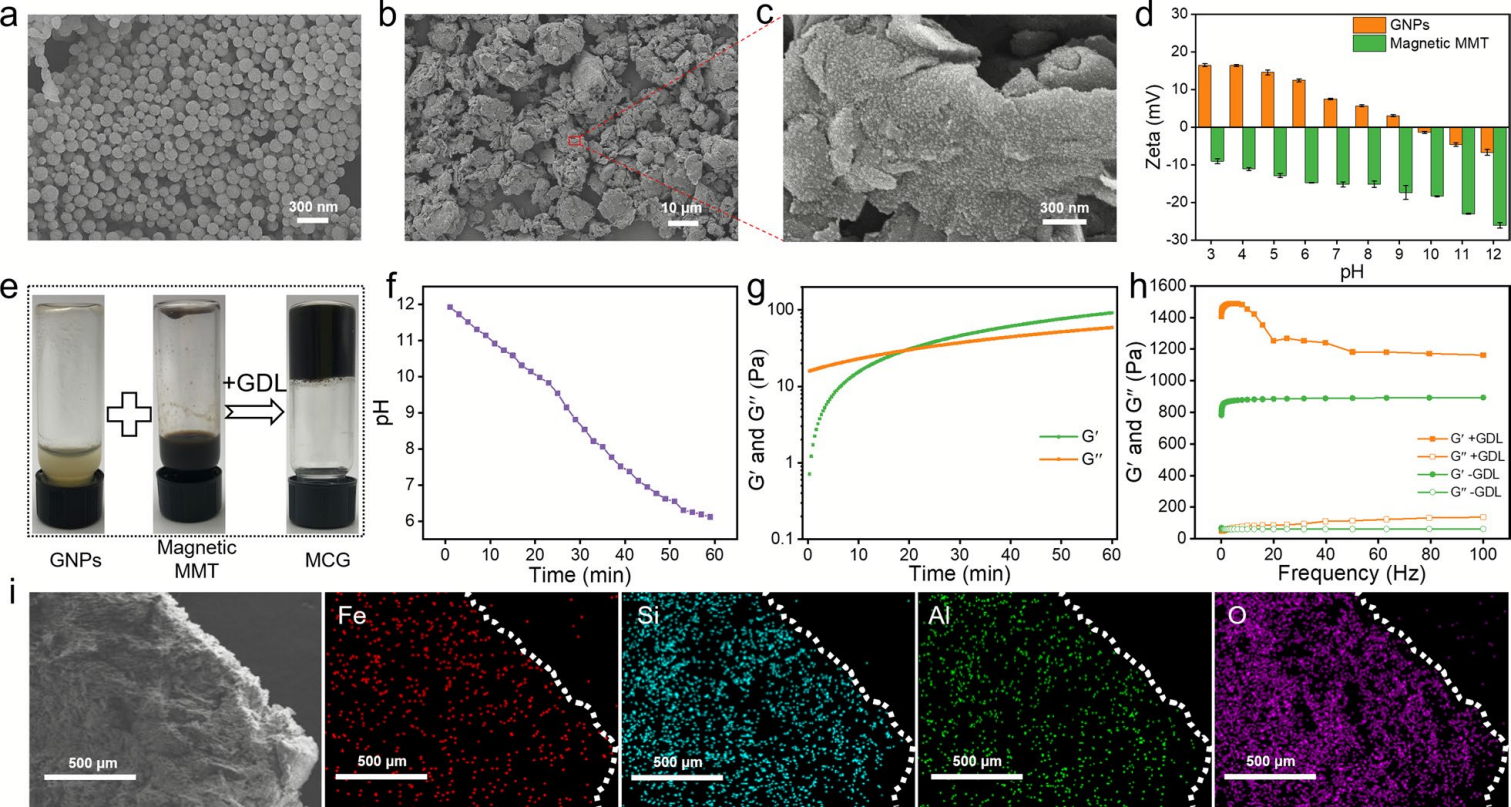
Preparation and characterization of MCG. Scanning electron images of **a** GNPs, and magnetic MMT at low (**b**) and high (**c**) magnification. **d** Zeta potential changes of GNPs and magnetic MMT at various pH values. **e** Images of colloidal particle dispersion and MCG after an inverted-vial test. **f** The pH change of colloidal system during decomposition of GDL. **g** Evolution of storage G′ and loss G′′ moduli during the MCG formation process. **h** The modulus of the pH-induced gel and directly mixed gel. **i** The element distribution of MCG

The physicochemical properties of the basic building blocks of the MCG were characterized. GNPs served as the positively charged building block, and the lyophilized GNPs were highly spherical (Fig. 1a). The hydrated particle size of GNPs and DOX@GNPs was approximately 300 nm, as measured by dynamic light scattering (DLS) (Additional file 1: Fig. S1). As shown in Additional file 1: Fig. S2 and S4a, MMT was irregularly shaped with a two-dimensional sheet structure, and the particle size of MMT was approximately 9 μm. The surface of pure MMT was relatively smooth. Then, iron oxide nanoparticles (IONs) were synthesized in situ on the surface of MMT according to our previous report on magnetic graphene oxide nanosheets [42]. As observed in the SEM images of Fig. 1b and c, the surface of magnetic MMT was relatively rough compared with pure MMT, and this homogeneous roughness indicated that IONs were synthesized uniformly in situ on the surface of the MMT. The X-ray diffraction (XRD) patterns also demonstrated that IONs were successfully synthesized (Additional file 1: Fig. S3). There was no significant change in the size and surface zeta potential of pure MMT after in situ magnetic modification (Additional file 1: Fig. S4). Due to the revealed charge-conversion property of GNPs, the assembly process of colloidal gel could be controlled by regulating the pH of the solution. As shown in Fig. 1d, the zeta potentials of GNPs and MMT were measured at different pH values. At an initial pH of 12, both the GNPs and magnetic MMTs exhibited negative charges, and thus, these colloidal particles could be uniformly mixed in aqueous solution. The gelation of the binary gel was driven by the pH. GDL was introduced as an acidifier, which was decomposed to gradually decrease the pH from 12 to 7 (below the isoelectric point of GNP at pH = 9). The tube-inversion test visually illustrated the gelation of MCG (Fig. 1e).

As the pH of the binary colloidal system decreased (Fig. 1f), the charge of GNPs gradually changed from negative to positive, and repulsion was no longer strong enough to prevent collision of the nanoparticles. The electrostatic attraction between GNPs and magnetic MMT was enhanced and eventually formed a network of interconnected particles. Rheological changes also accompanied the formation of the colloidal gel. Rheological data showed the process of the change from liquid to solid (Fig. 1g). To verify the effect of the charge-conversion property of GNPs, a control test was carried out, in which two kinds of particles with opposite charges were directly and simply mixed when the pH was equal to 7, the obtained hydrogel was named as MCG without GDL (MCG_−GDL_). As shown in Fig. 1h, MCG_−GDL_ exhibited a significantly lower stiffness than MCG_+ GDL_. These results indicated that regulating pH (from alkaline to neutral) was attributed to a more uniform distribution of particles in the preparation process of MCG. The element scanning mapping of the MCG (Fig. 1i) showed the uniform distribution of Fe, Si, Al and O in the gel, illustrating the uniform distribution of the two building blocks. Furthermore, rheological tests were performed on MCG with different solid contents (Additional file 1: Figs. S5 and S6), and considering the magnetic heating properties and elasticity needed for interventional thermotherapy, MCG (10 w/v %) with a mass ratio value of 1 was selected for the subsequent experiments.

### Stability enhancement and injectability of MCG

**Fig. 2:**
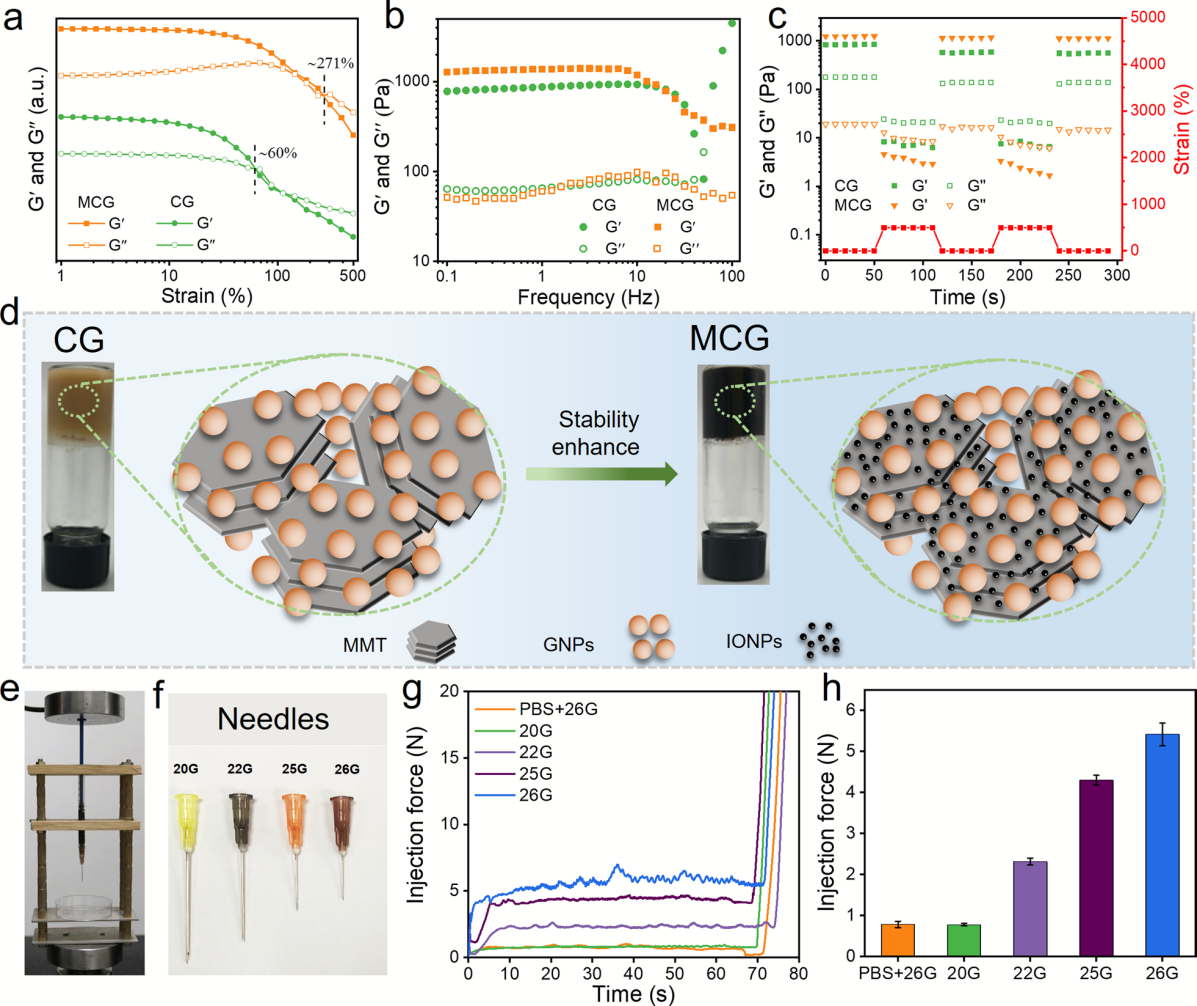
Stability enhancement and injectability of MCG. **a** G′ and G′′ versus frequency and **b** G′ and G′′ on strain amplitude sweep for CG, and MCG at 25 °C. **c** G′ and G′′ for CG and MCG with alternating shear strains of low (1%)—high (500%)—low (1%) strain at the frequency of 1 Hz. **d** Schematic diagram of CG and MCG microstructure comparison. **e** The homemade injection device. **f** The digital images of needles with different sizes. **g** The injection force of MCG (10 w/v %) with different needles. **h** The injection force of MCG (10 w/v %) in the first minute with different needles

According to a previous report [43], higher friction led to an enhanced mechanical property of bioinspired ceramics. In this study, the surface roughness of magnetic MMT was increased after the loading of IONs onto MMT, which enhanced the friction between the MMTs and GNPs. Standard rheological tests were performed to investigate the influence of IONs on the rheological properties of hydrogels. MMTs with different Fe contents (0, 5, 10, 15 and 20% wt.) were synthesized and served as the building blocks of MCG. As shown in Fig. 2a and Additional file 1: Fig. S7a–f, the critical strain value of MCG varied with the Fe content. When the Fe content was 15%, the critical strain value reached its maximum (~ 271%), which was significantly higher than that of CG (~ 60%) and the other samples. When the frequency varied from 0.1 Hz to 100 Hz, the MCG system remained stable, whereas the CG system collapsed when the frequency reached approximately 50 Hz (Fig. 2b). The shear-thinning properties of MCG with different Fe contents showed that all the gels had excellent injection ability (Additional file 1: Fig. S8). Moreover, dynamic strain tests (from strain 0.5–500%) were performed on MCG and CG (Fig. 2c). The results indicated that the modulus of both gels could recover rapidly, and the G’ of MCG at 0.5% strain was restored to 99% of the initial modulus, while the G′ of CG at 0.5% strain was restored only to 80%. As a result, the introduction of magnetic nanoparticles into the colloidal gel further contributed to the enhancement of stability and self-healing properties (Fig. 2d). In addition, the injection forces of MCG corresponding to needles with different diameters were recorded in real-time using a universal testing machine (Fig. 2e and f and Additional file 1: Fig. S9). As shown in Fig. 2g, MCG was easily extruded using syringes with very little injection force until the plunger reached the nozzle, as evidenced by a sharp increase in the injection force. Using a 26G needle, the injection force was as low as 5 N (Fig. 2h), which indicated that MCG could meet the requirements for interventional injection into HCC-located regions.

### Magnetic hyperthermia performance

**Fig. 3:**
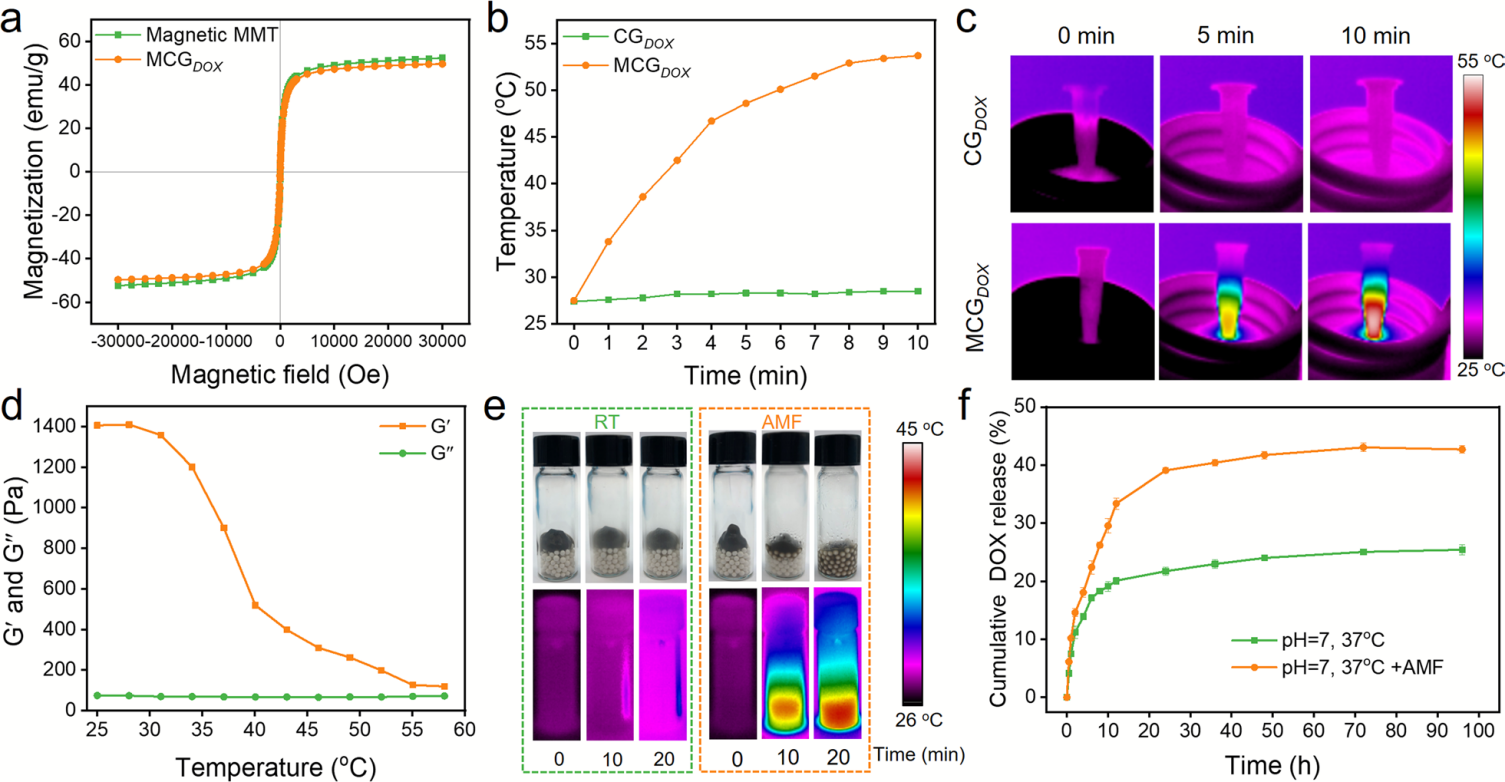
Magnetic hyperthermia performance. **a** Magnetization curve of magnetic MMT and MCG_*DOX*_. **b** MCG_*DOX*_ and CG_*DOX*_ temperature change curve and **c** Corresponding IR thermal images under AMF (30 kA/m). **d** The temperature-dependent variation in G′ and G′′ of MCG_*DOX*_. **e** The self-conformal behavior of MCG_*DOX*_ under AMF and room temperature. **f** The cumulative drug release curve of MCG_*DOX*_ with or without AMF application

MCG_*DOX*_ was prepared with GNPs loaded with DOX and magnetic MMT by electrostatic attraction. As shown in Fig. 3a, the hysteresis loops indicated the superparamagnetic behavior of both magnetic MMT and MCG_*DOX*_, and the saturation magnetization of MCG_*DOX*_ was 55 emu/g. The magnetic heating effects of MCG_*DOX*_ and CG_*DOX*_ were evaluated after exposure to AMF (Fig. 3b and c). The temperature of MCG_*DOX*_ increased to approximately 53 °C in 10 min, and this hyperthermic effect was expected to enable the effective killing of cancer cells. The temperature increase cycle illustrated the magnetic heating stability of MCG_*DOX*_ (Additional file 1: Fig. S10). Rheological experiments were performed to reveal the effect of temperature on the modulus of MCG_*DOX*_. As shown in Fig. 3d, the storage modulus decreased gradually with increasing temperature, while no significant change was observed in the viscous modulus, clearly indicating that the fluidity was thermosensitive and increased as the temperature increased. To illustrate the fluidity enhancement of MCG_*DOX*_ under magnetothermal stimulation, MCG_*DOX*_ was injected into two glass bottles containing zirconium beads [18]. Both of the bottles were placed at room temperature, and one of them was placed under an AMF (Fig. 3e). After 20 min, MCG_*DOX*_ remained on the surface of the zirconium beads, and little infusion occurred. In contrast, due to the thermosensitivity of the fluidity, MCG_*DOX*_ displayed significant self-conformal behavior and filled all of the bead space within 20 min under AMF exposure. In addition to enhancing the fluidity of MCG_*DOX*_, magnetic heating helped to accelerate drug release from MCG. The drug release curve of MCG_*DOX*_ was detected by a dialysis method (PBS, pH = 7.4). MCG_*DOX*_ released a dramatic amount of DOX with the help of AMF, approximately twice the release of MCG_*DOX*_ in the absence of AMF, indicating that the AMF stimulus accelerated the release of DOX from MCG_*DOX*_ (Fig. 3f).

### In vitro and in vivo biocompatible evaluation

**Fig. 4:**
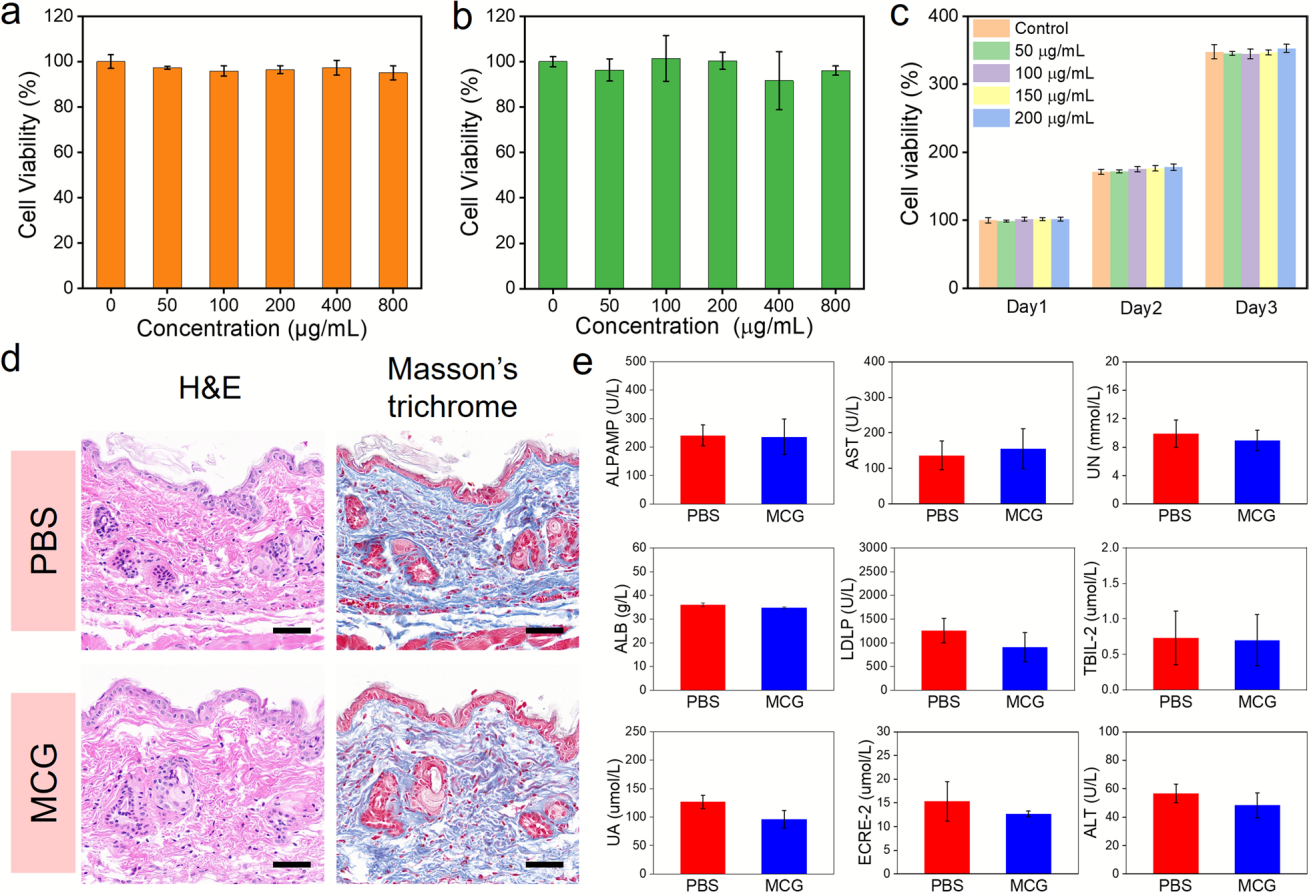
In vitro and in vivo biocompatible evaluation. The cell viability of NIH-3T3 incubated with various concentrations of **a** GNPs, **b** magnetic MMT and **c** MCG. **d** MCG embedded tissue and healthy tissue examination by Masson’s trichrome and H&E staining (Scale bar: 50 μm). **e** The hematological analysis of mice 2 weeks after injection of PBS and MCG

Two components (magnetic MMTs and GNPs) and MCG were cocultured with NIH-3T3 cells at gradient concentrations. The observation showed that the cell viability was not decreased significantly (Fig. 4a–c), and the cells in contact with the MCG could be differentiated normally after 3 days, demonstrating the good biocompatibility of MCG. Furthermore, typical Masson’s trichrome and hematoxylin & eosin (H&E) tissue staining methods were used to analyze the pathological changes at the MCG implantation site. As shown in Fig. 4d, there were few inflammatory factors in the tissue and no pathological changes. The hematological analysis data (Fig. 4e) further revealed that the mice implanted with MCG showed no significant pathological changes compared to healthy mice. Therefore, in vitro and in vivo experiments confirmed that MCG has excellent biocompatibility, indicating the great potential for implanted manipulation into postoperative tumor areas without damaging healthy tissues.

### Evaluation of hemostasis and tumor therapy performance after tumor surgery

**Fig. 5:**
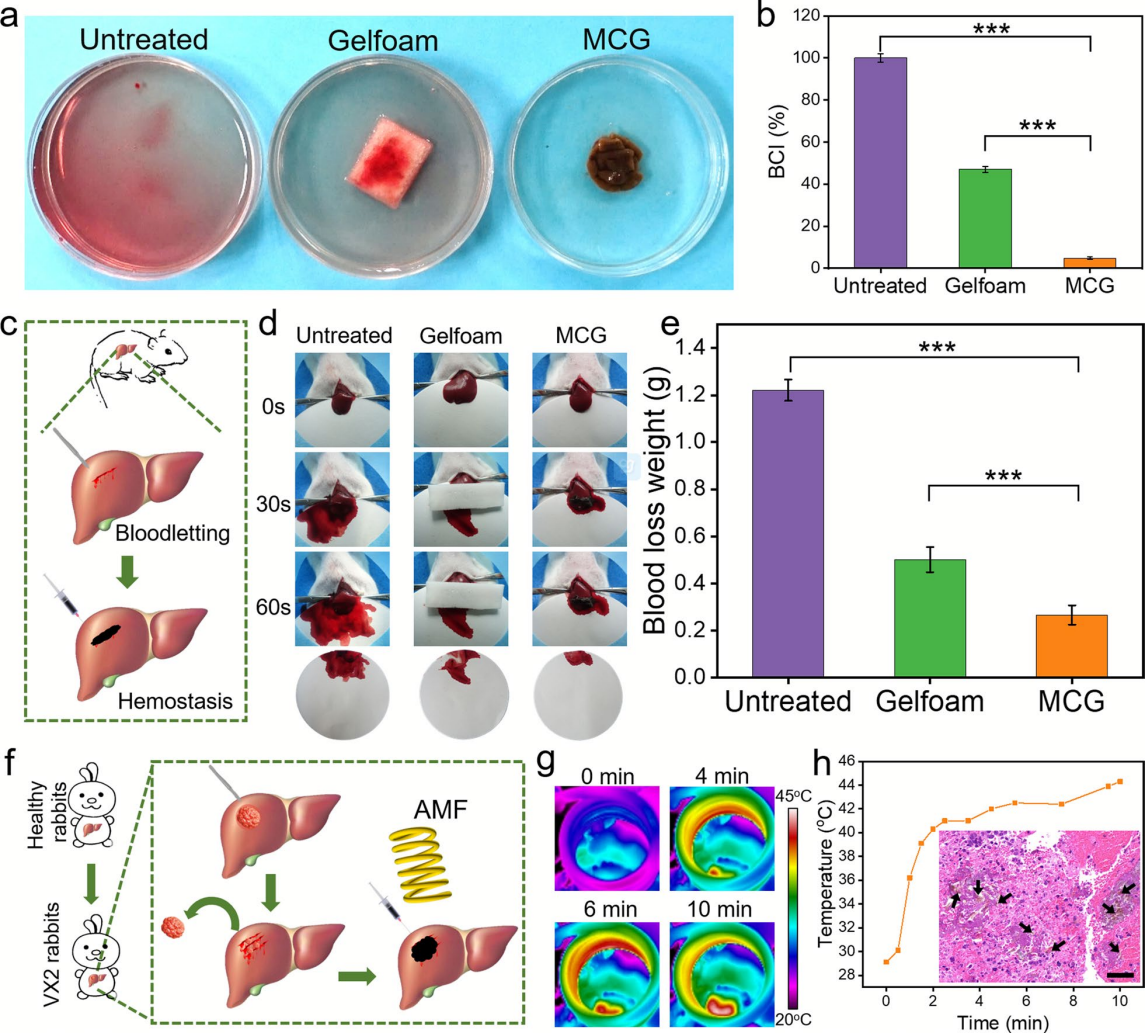
Evaluation of hemostasis and tumor therapy performance after tumor surgery. **a** In vitro dynamic whole-blood-clotting evaluation of the MCG. **b** BCI value of MCG, Gelfoam and untreated groups. **c** Schematic illustration of the hemostasis by using the MCG in rat liver hemorrhage model. In vivo hemostatic evaluation was performed by recording. **d** Photographs of hemostasis and blood loss in wounds with different treatments. **e** The quantitative blood loss weight after different treatments. **f** The schematic of VX2 liver cancer rabbit model was established to achieve hemostasis after surgery and synergistic therapeutic effects of the MCG. **g** Temperature of the surgical site monitored by Fluke infrared thermal imager. **h** The temperature rises curve of the tumor site. (Inset: H&E staining analysis of tumor sections harvested from tumor sites after treatment. The black arrows represent the MCG pieces. Scale bar: 50 μm) (**p < 0.01, ***p < 0.001)

An in vitro dynamic whole-blood-clotting test was performed to evaluate the blood-clotting index (BCI) [44], and a lower BCI indicated a higher clotting rate. As shown in Fig. 5a and b, obvious hemolysis was observed in the untreated and commercial gelfoam groups, but there was almost no hemolysis in the MCG group. Moreover, the lowest BCI value in the MCG group confirmed the potential hemostatic ability of MCG. The hemostatic capability of MCG was investigated in vivo by using a liver bleeding model in rats (Fig. 5c). As shown in Fig. 5d, liver bleeding stopped instantly after MCG injection into the surgical wound, whereas the gelfoam and untreated groups showed visible bleeding after 60 s. As shown in Fig. 5e, the blood loss in the MCG group (0.275 ± 0.040 g) was less than that in the untreated group (1.221 ± 0.045 g). There was also a noticeable reduction in blood loss using MCG compared with the commercial gelfoam group (0.501 ± 0.053 g), which indicated the remarkable hemostasis efficiency of MCG. The effective hemostatic ability of MCG should be attributed to two aspects. On the one hand, due to the good fluidity, the MCG could penetrate the wound gap to completely seal the lesion and serve as a physical barrier to stop the bleeding. As shown in Additional file 1: Fig. S11, H&E images of damaged liver sections showed that the remaining hydrogel was trapped in the wound gap after MCG treatment. On the other hand, the negatively charged magnetic MMT surface reinforced the stimulation of blood cells and improved the coagulation capacity of MCG (Additional file 1: Fig. S12) [35, 45]. The rabbit VX2 liver tumor model was established to verify the postoperative hemostasis and therapeutic effects of MCG (Fig. 5f). The primary liver tumor was surgically removed, followed by injecting MCG onto the surface of the hepatic operative wound. MCG could seal the wound sutureless and stop bleeding instantly (Additional file 1: Fig. S13). Thereafter, the operative wound site was placed under an AMF for 10 min. The temperature of the MCG was monitored by an infrared thermal imager (Fig. 5g), and the temperature in the tumor site rose quickly after the injection of MCG, finally reached to 44.3 °C (Fig. 5 h). The heating effect is sufficient to inhibit tumor growth. The H&E staining results demonstrated that the residual HCC on the rabbit liver was effectively killed (inset of Fig. 5h). Moreover, Additional file 1: Fig. S14 showed MCGs were attached to liver and tumor defects and still stayed on the postoperative wound before and after applying AMF, illustrating that the MCG could be adapted to fill the irregular tissue defects. All of these results revealed that the MCG developed in the current research exhibits strong hemostatic ability and has potential as adjuvant therapy after HCC surgery.

### Integration of magnetic hyperthermia and chemotherapy prevents tumor recurrence

**Fig. 6:**
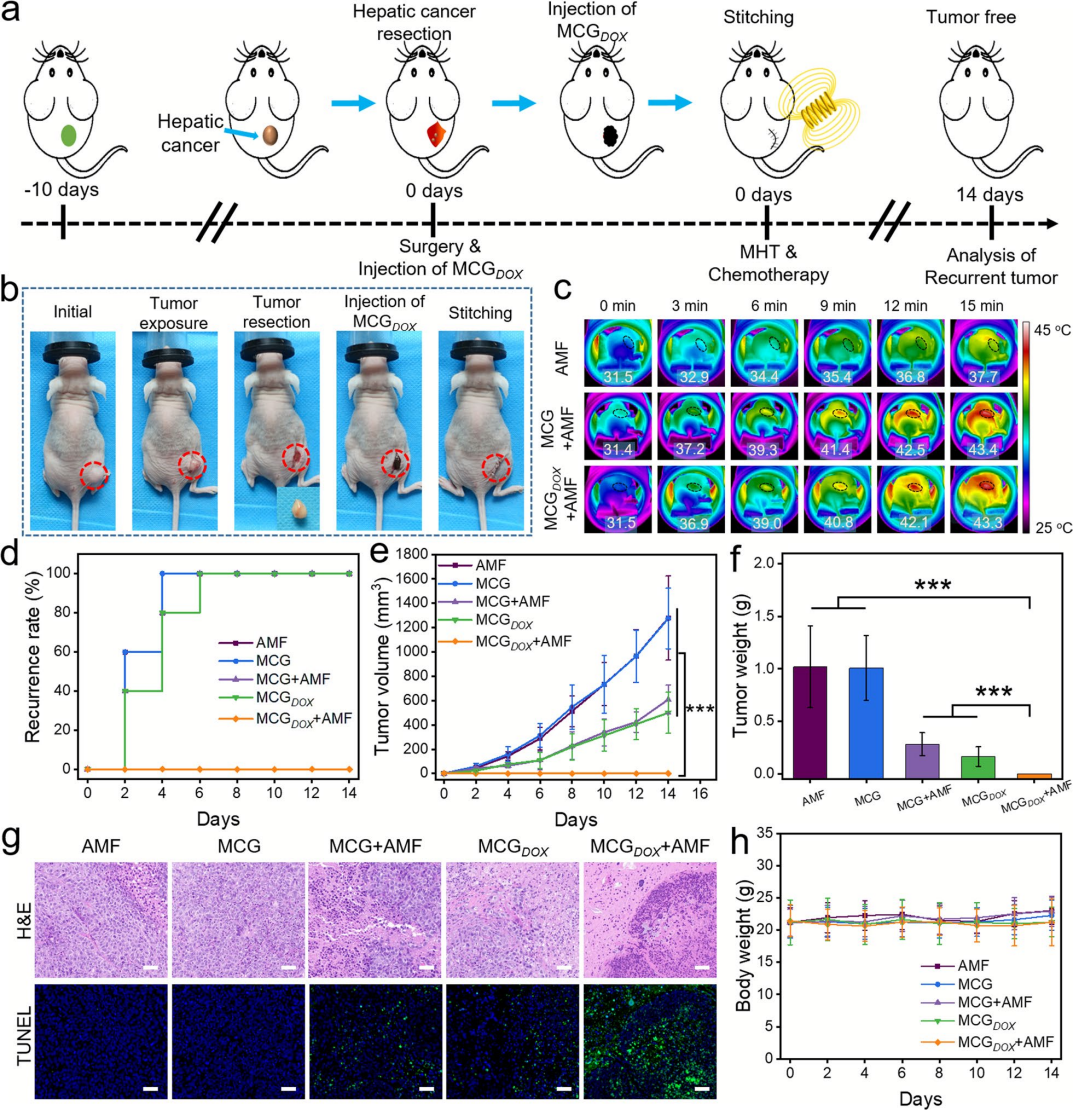
Integration of magnetic hyperthermia and chemotherapy prevents tumor recurrence. **a** Schematic illustration of MCG_*DOX*_ prevention of tumor recurrence using a HepG2 tumor-bearing mouse model. **b** Representative photos during tumor resection. **c** IR thermal images of mice with the postoperative site of tumor injected by MCG, and MCG_*DOX*_ under AMF (H = 30 kA/m, f = 312 kHz) for 15 min. **d** The recurrence rate of mice bearing hepatic tumor after several treatments. **e** The hepatic tumor growth curves and **f** mice tumor weight in each group. **g** H&E staining and TUNEL staining analysis of the surgical area in all groups at 14th day (Scale bar: 50 μm). **h** Body weight curve of each group in 14 days. (**p < 0.01, ***p < 0.001)

Surgical resection is the major treatment strategy for various cancers. However, the very high local recurrence rate of cancer after surgical resection remains a clinically fatal problem [46–48]. Inspired by the in vitro magnetic hyperthermia effect and controlled drug release of MCG_*DOX*_, MCG_*DOX*_ was expected to effectively inhibit locoregional tumor recurrence after resection. Subsequently, a HepG2 tumor-bearing mouse model was used to evaluate the efficacy of MCG_*DOX*_ in preventing tumor recurrence (Fig. 6a). The photos in Fig. 6b showed the process of the tumor surgical operation. All of the mice were randomly grouped into five groups, and the administered materials were injected into the tumor bed in situ by a syringe. All the mice in the AMF group, MCG + AMF group and MCG_*DOX*_+AMF group were exposed to AMF for 15 min. As shown in Fig. 6c, the temperatures of tumor regions in both the MCG + AMF group and MCG_*DOX*_+AMF group increased to ~ 42 °C within 12 min, and this hyperthermic effect was able to restrain tumor recurrence. After these treatments, the tumor recurrence rate curve within 14 days after treatment was plotted (Fig. 6d), which showed that the mice treated with MCG_*DOX*_+AMF had the lowest tumor recurrence rates (0%). Thereafter, the tumor growth curve of the five groups in 14 days indicated that only the magnetic hyperthermia and chemotherapy groups showed efficient tumor suppression effects (Fig. 6e). Photographs of mice and the excised tumors in each group before and 14 days after treatment are shown in Fig. 6f and Additional file 1: Figs. S15, S16, illustrating the markedly enlarged tumors of mice in the AMF and MCG groups. Both the MCG + AMF and MCG_*DOX*_ groups showed obvious inhibition of the tumor. By comparison, the strategy of MCG_*DOX*_ injection with AMF exposure, combining MHT and chemotherapy, achieved a remarkable suppression recurrence effect with no tumor growth signal. As illustrated in Fig. 6g, H&E staining and TUNEL staining of the recurrent tumors from the MCG_*DOX*_+AMF group further proved that the intact region of tumor tissue appeared as distinct necrosis and that the tumor cells were destroyed, whereas the other groups still largely retained an abundance of tumor cells. Furthermore, as shown in Fig. 6h and Additional file 1: Fig. S17, no obvious weight changes were found in any group, and no observed pathological changes in these organs were found after local thermo-chemotherapy using MCG_*DOX*_, suggesting the high safety of the magnetic hydrogel-based therapeutic approach for preventing cancer recurrence. The above results demonstrated that injectable MCG_*DOX*_ exhibits synergistic antitumor effects as a highly effective postoperative adjuvant therapy strategy.

### Interventional thermotherapy of HCC

**Fig. 7:**
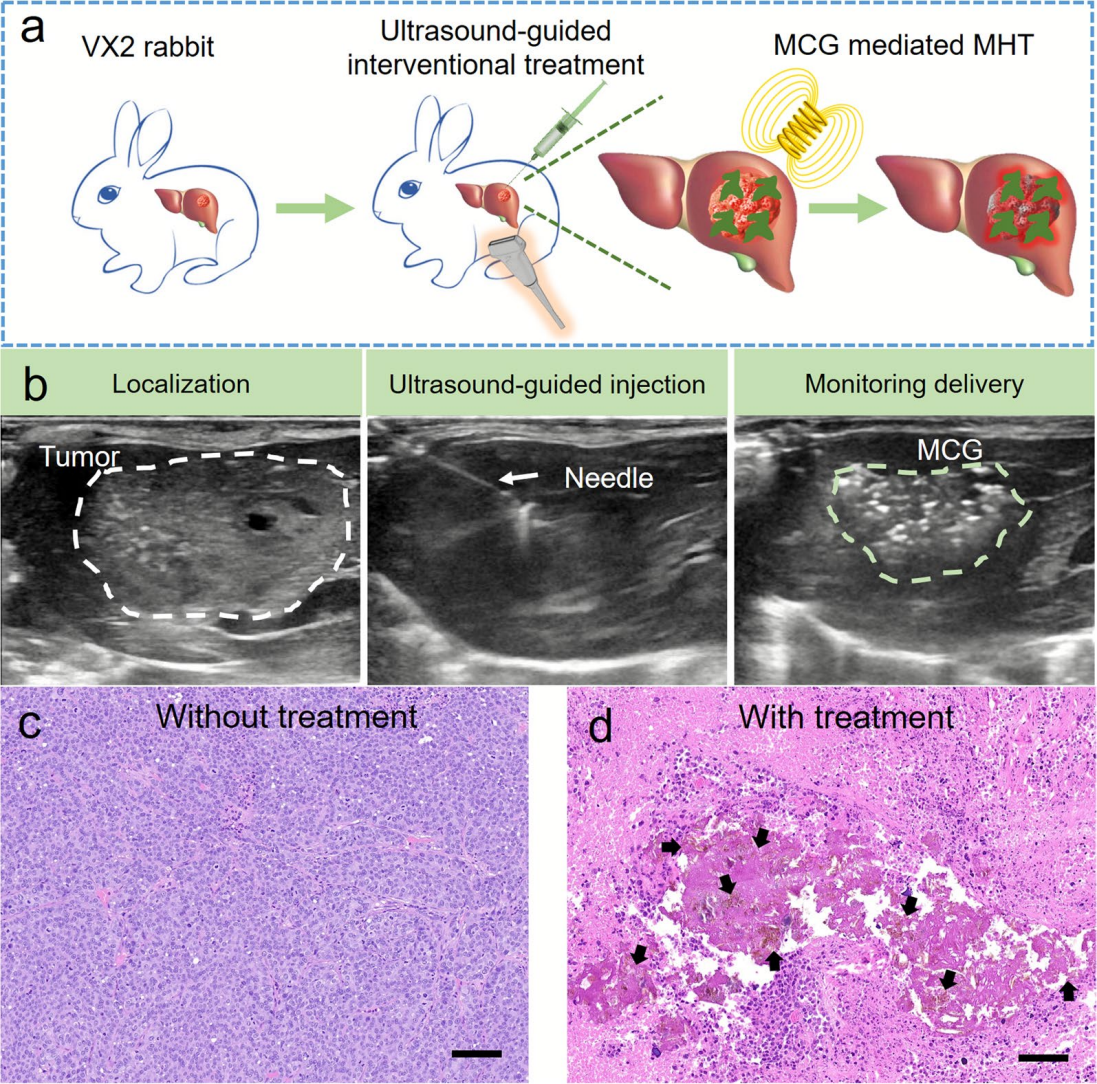
Interventional thermotherapy of HCC. **a** The diagram of ultrasound-guided percutaneous puncture combined with magnetocaloric therapy in the treatment of deep tumor. The MCG was injected into the VX2 tumor by percutaneous puncture under ultrasonographic guidance, and then the rabbits received MHT. **b** Ultrasound guided injection of MCG. The white dash line and green dash line indicated VX2 tumor and MCG in tumor, respectively. H&E staining analysis of tumor sections harvested from tumor sites before treatment (**c**) and after treatment (**d**). The black arrows represent the MCG pieces (scale bar: 100 μm)

As a minimal invasive therapy, MCG-induced interventional magnetic hyperthermia under ultrasound guidance could efficiently treat postoperative recurrence when the secondary surgical resection was not recommended. As shown in Fig. 7a, MCG was injected into the VX2 tumor by percutaneous puncture under ultrasonographic guidance, and then the rabbit received MHT. As shown in Fig. 7b, the VX2 model was monitored and localized by ultrasound imaging, followed by ultrasound imaging-guided interventional injection of MCG into the tumor region in the liver. After magnetic hyperthermia treatment for 15 min, the tumor tissues were collected for H&E staining analysis. As shown in Fig. 7c and d, compared with the untreated tumor tissue, MCG ingredients were observed in the treated tumor tissue, and significant cell apoptosis occurred around the gel debris. These results demonstrated that MCG can provide an available interventional MHT to treat postoperative recurrence.

## Conclusions

In summary, we have developed a DOX-loaded injectable MCG that combines hemostasis, MHT and chemotherapy to prevent tumor recurrence after the resection of HCC. Magnetic MMTs were connected with amphiprotic GNPs to formulate a stable colloidal network. By regulating the ratio between two building blocks, composite colloidal gels were produced with good viscoelasticity and self-healing performance. The introduction of IONs improved the stability of the MCG network and endowed MCG with a magnetic heating effect. In vivo experiments showed that MCG promoted wound hemostasis quickly and effectively after tumor surgery. Additionally, MCG_*DOX*_-mediated MHT and chemotherapy had a remarkable effect in preventing the postoperative recurrence of the tumor. On VX2 tumor rabbits, the successful implementation of ultrasound-guided interventional MHT further confirmed the alternative minimal invasive therapeutics to treat the postoperative recurrence by MCG. The current research introduced exciting opportunities for colloidal gels with a particulate network as a postoperative adjuvant therapy for tumors.

## Supplementary Information

**Additional** **file** **1:** **Figure S1.** Particle size of (a) GNPs and (b) DOX@GNPs at pH 7. **Figure S2.** (a-b) SEM images of pure MMT. **Figure S3.** X-ray diffraction patterns of MMT, magnetic MMT, GNPs and MCG. **Figure S4.** (a) Particle size of magnetic MMT and MMT. (b) Zeta potential of magnetic MMT and MMT at pH 7. **Figure S5.** (a) The elastic (G′) and viscous (G′′) moduli of the GNPs and MMT suspension liquid. (b) Frequency dependence of G′ and G′′ of MCG with different solid contents. (c) Loss factor (tan δ) of MCG with different solid contents. (d) Shear-thinning behaviors of MCG with different solid contents. **Figure S6.** (a) Loss factor (tanδ) and (b) frequency dependence of MCG with different weight ratios of magnetic MMT to GNPs. **Figure S7.** The G′ and G′′ on strain amplitude sweep for MCGs with different mass fractions of Fe (0-20%). **Figure S8.** Shear-thinning behaviors of MMTs with the different mass fractions of Fe. **Figure S9.** Table of needle dimensions. **Figure S10.** The magnetic hyperthermia conversion cycling test of MCG. **Figure S11.** H&E images of damaged liver section. **Figure S12.** The SEM of that MCG leads to the formation of fibrin network. **Figure S13.** Photographs of hemostasis after HCC resection on a rabbit. **Figure S14.** MCGs were attached to liver (a) or tumor (b) defects before and after applying AMF. **Figure S15.** The digital images of excised HepG2 tumor after treatment at 14th day. **Figure S16.** Photographs of mice from each group at 0 day and 14th day. **Figure S17.** Representative H&E images of major organs of representative mice from the healthy group and MCG_*DOX*_+AMF group.

## Data Availability

Most of the datasets supporting the conclusions of this study are included within the manuscript and the additional files. The datasets used or analyzed during the study are available on reasonable request.

## Abbreviations

MCGs: Magnetic colloidal gels
IONs: Iron oxide nanoparticles
HCC: Hepatocellular carcinoma
NIR: Near-infrared light
AMF: Alternating magnetic field
GDL: D-(+)-gluconic acid δ-lactone
MHT: Magnetic hyperthermia therapy
MMT: Montmorillonite
XRD: X-ray diffraction
PSS: Sodium polystyrene sulfonate
GNPs: Gelatin nanoparticles
BCI: Blood-clotting index
DOX: Doxorubicin
DLS: Dynamic light scattering
H&E: Hematoxylin and eosin
PBS: Phosphate buffered saline
SEM: Scanning electron microscope
