# Supplementary material for: Injectable magnetic montmorillonite colloidal gel for the postoperative treatment of hepatocellular carcinoma
Source: J Nanobiotechnology. 2022 Aug 19;20:381. doi: 10.1186/s12951-022-01559-7 (PMC9392261; doi:10.1186/s12951-022-01559-7)
Supplement: Supplementary file 1 — Additional file 1: Figure S1. Particle size of (a) GNPs and (b) DOX@GNPs at pH 7. Figure S2. (a-b) SEM images of pure MMT. Figure S3. X-ray diffraction patterns of MMT, magnetic MMT, GNPs and MCG. Figure S4. (a) Particle size of magnetic MMT and MMT. (b) Zeta potential of magnetic MMT and MMT at pH 7. Figure S5. (a) The elastic (G′) and viscous (G′′) moduli of the GNPs and MMT suspension liquid. (b) Frequency dependence of G′ and G′′ of MCG with different solid contents. (c) Loss factor (tan δ) of MCG with different solid contents. (d) Shear-thinning behaviors of MCG with different solid contents. Figure S6. (a) Loss factor (tanδ) and (b) frequency dependence of MCG with different weight ratios of magnetic MMT to GNPs. Figure S7. The G′ and G′′ on strain amplitude sweep for MCGs with different mass fractions of Fe (0-20%). Figure S8. Shear-thinning behaviors of MMTs with the different mass fractions of Fe. Figure S9. Table of needle dimensions. Figure S10. The magnetic hyperthermia conversion cycling test of MCG. Figure S11. H&E images of damaged liver section. Figure S12. The SEM of that MCG leads to the formation of fibrin network. Figure S13. Photographs of hemostasis after HCC resection on a rabbit. Figure S14. MCGs were attached to liver (a) or tumor (b) defects before and after applying AMF. Figure S15. The digital images of excised HepG2 tumor after treatment at 14th day. Figure S16. Photographs of mice from each group at 0 day and 14th day. Figure S17. Representative H&E images of major organs of representative mice from the healthy group and MCGDOX+AMF group. [file 12951_2022_1559_MOESM1_ESM.docx]

**Supporting Information**

**Injectable Magnetic Montmorillonite Colloidal Gel for the Postoperative Treatment of Hepatocellular Carcinoma**

Sheng Chen^1†^, Yonghong Song^1†^, Xu Yan^1†^, Liang Dong^2,4*^, Yunjun Xu^2^, Shouhu Xuan^2^, Quan Shu^2^, Baoqiang Cao^3^, Jinglong Hu^3^, Hanye Xing^1^, Wenshu Wu^1^, Zhengbao Zha^1*^ and Yang Lu^1*^

^*^Correspondence:

yanglu@hfut.edu.cn; dldisc@ustc.edu.cn; zbzha@hfut.edu.cn

^†^ Sheng Chen, Yonghong Song and Xu Yan contributed equally to this work.

^1^ Anhui Key Laboratory of Advanced Catalytic Materials and Reaction Engineering, School of Chemistry and Chemical Engineering, School of Food and Biological Engineering, Hefei University of Technology, Hefei 230009, China.

^2^ Division of Nanomaterials & Chemistry, Hefei National Laboratory for Physical Sciences at the Microscale, Department of Modern Mechanics, Department of Radiology, The First Affiliated Hospital of University of Science and Technology of China, University of Science and Technology of China, Hefei 230026, China.

^3^ Department of General Surgery, Department of Ultrasonics, Department of Interventional Radiology, Anhui No.2 Provincial People’s Hospital, Hefei, Anhui, 230041, China.

^4^ The Cancer Hospital of the University of Chinese Academy of Sciences (Zhejiang Cancer Hospital), Institute of Basic Medicine and Cancer (IBMC), Chinese Academy of Sciences, Hangzhou, Zhejiang 310022, China.

Full list of author information is available at the end of the article.

**
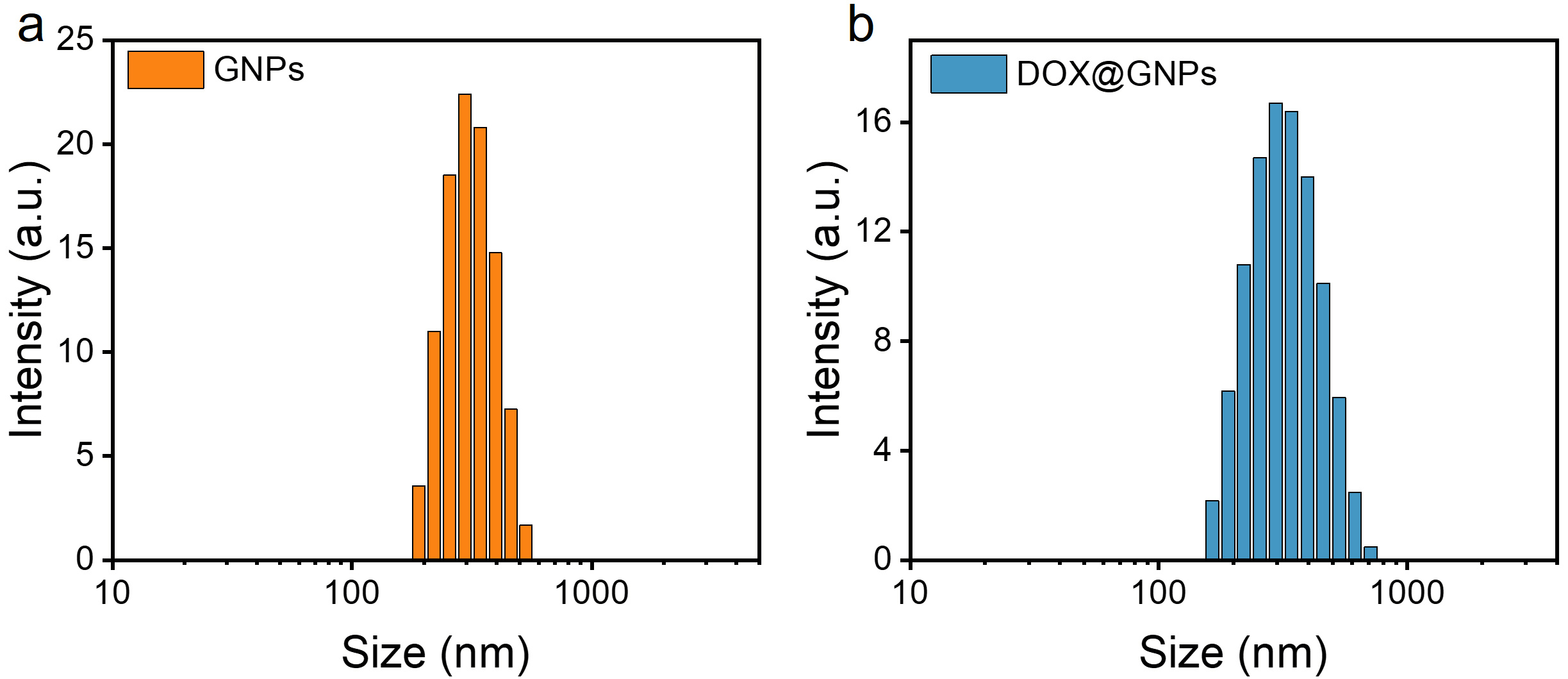
**

**Fig. S1** Particle size of (**a**) gelatin nanoparticles (GNPs) and (**b**) DOX loaded gelatin nanoparticles (DOX@GNPs) at pH 7.


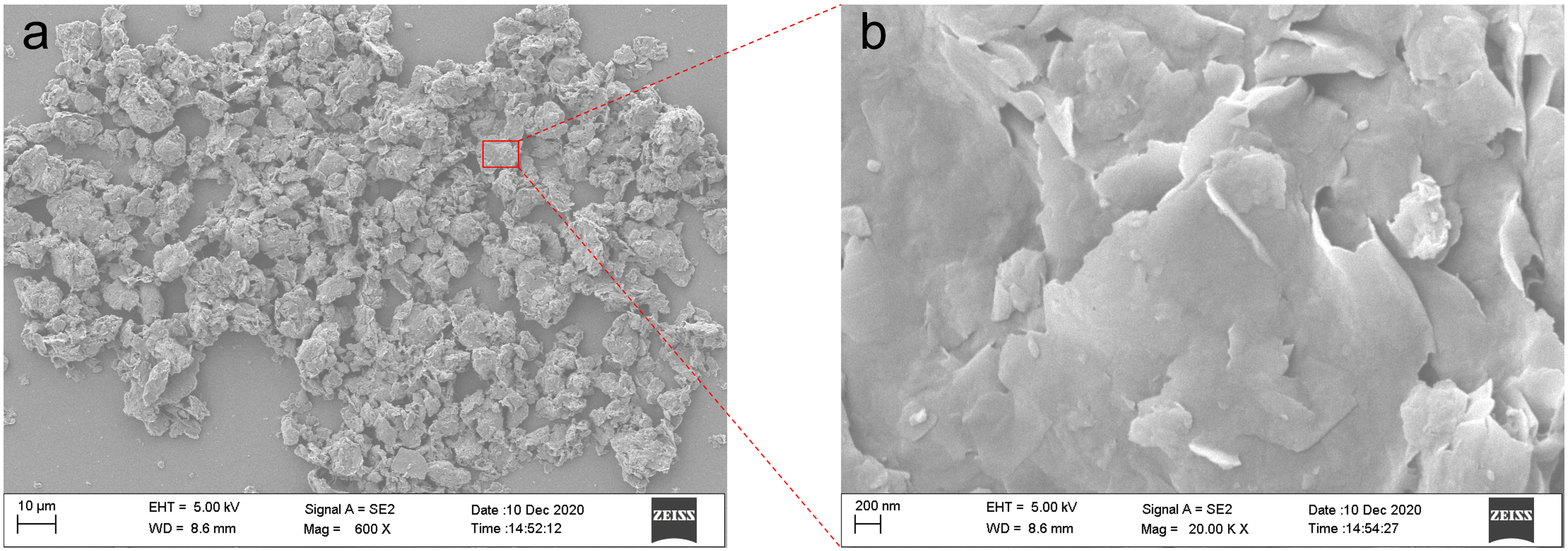


**Fig. S2** (**a-b**) SEM images of pure montmorillonite (MMT).


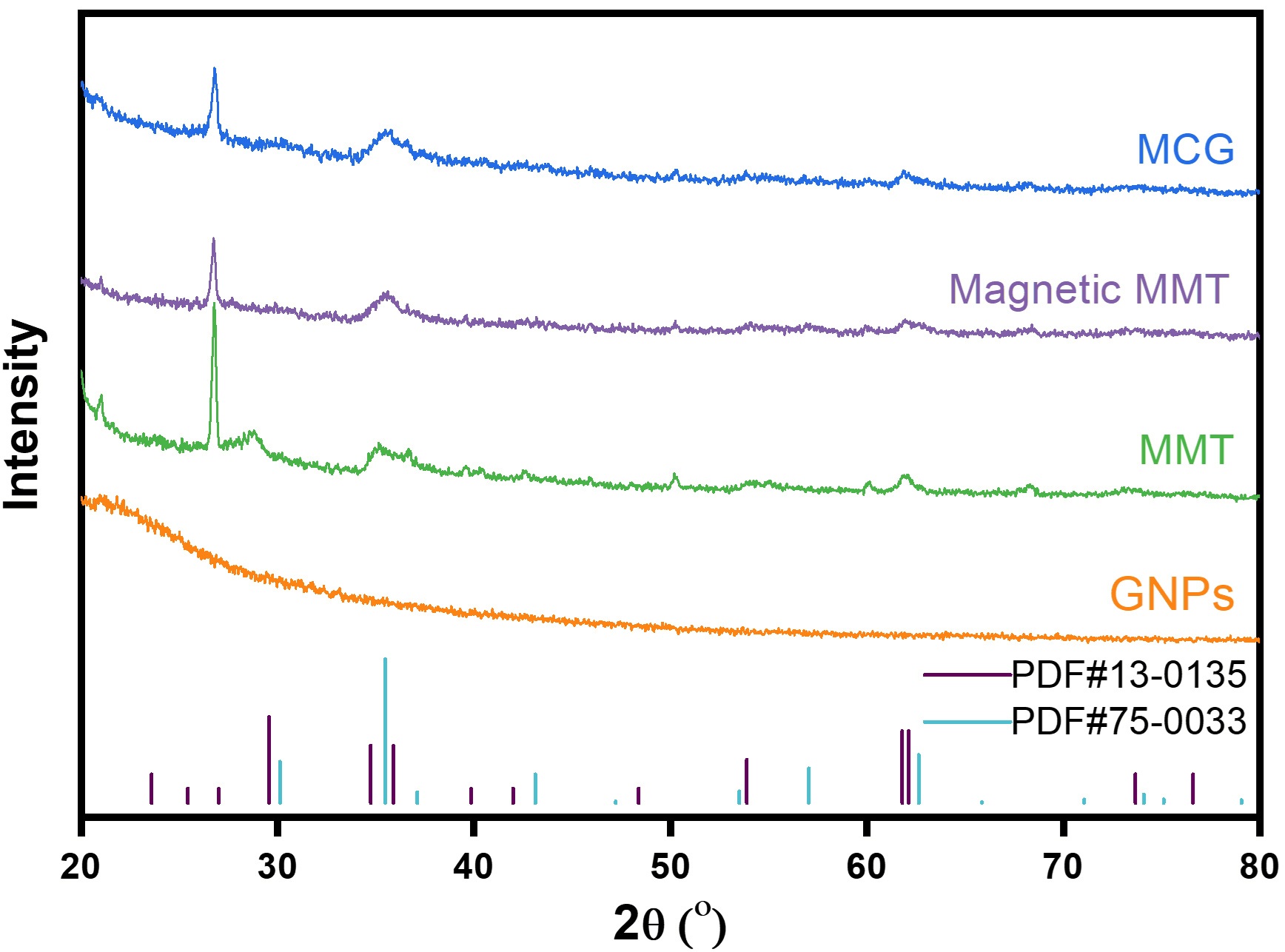


**Fig. S3** X-ray diffraction patterns of MMT, magnetic MMT, GNPs and MCG.


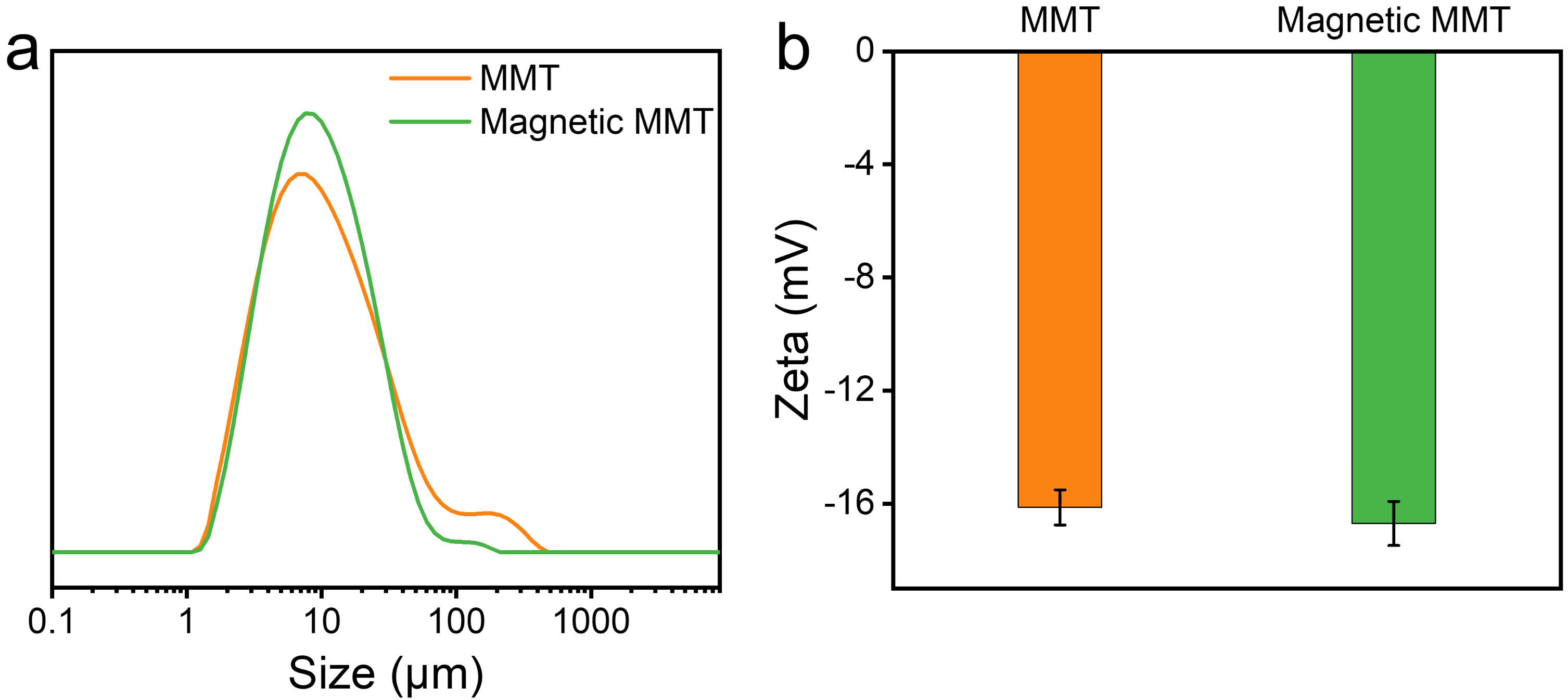


**Fig. S4** (**a**) Particle size of magnetic MMT (9.199 μm) and MMT (9.026 μm) at pH 7. (**b**) Zeta potential of magnetic MMT (-16.1 mV) and MMT (-16.7 mV) at pH 7.


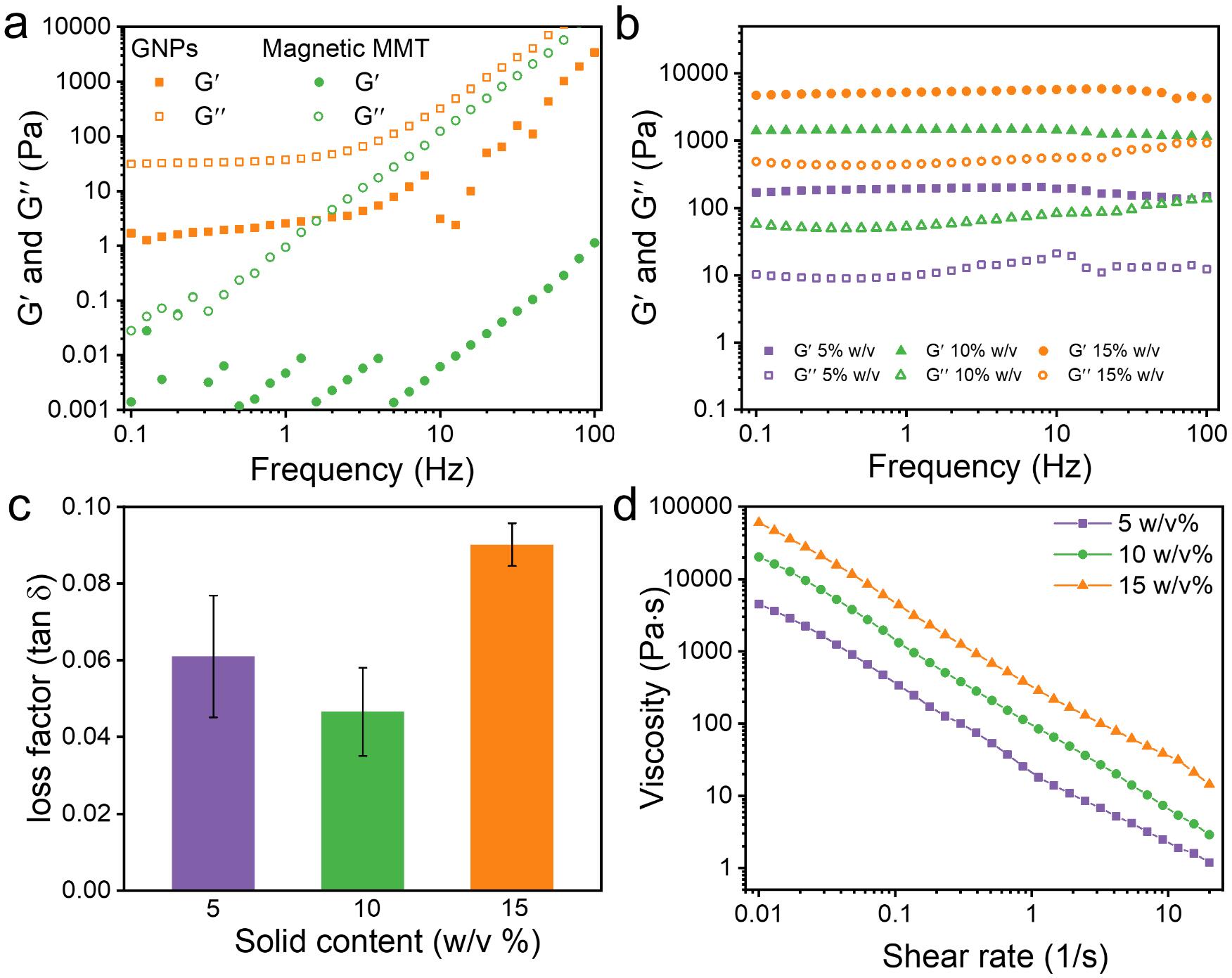


**Fig. S5** (**a**) The elastic (G′) and viscous (G′′) moduli of the GNPs and MMT suspension liquid. (**b**) Frequency dependence of G′ and G′′ of MCG with different solid contents. (**c**) Loss factor (tan δ) of MCG with different solid contents. (**d**) Shear-thinning behaviors of MCG with different solid contents.


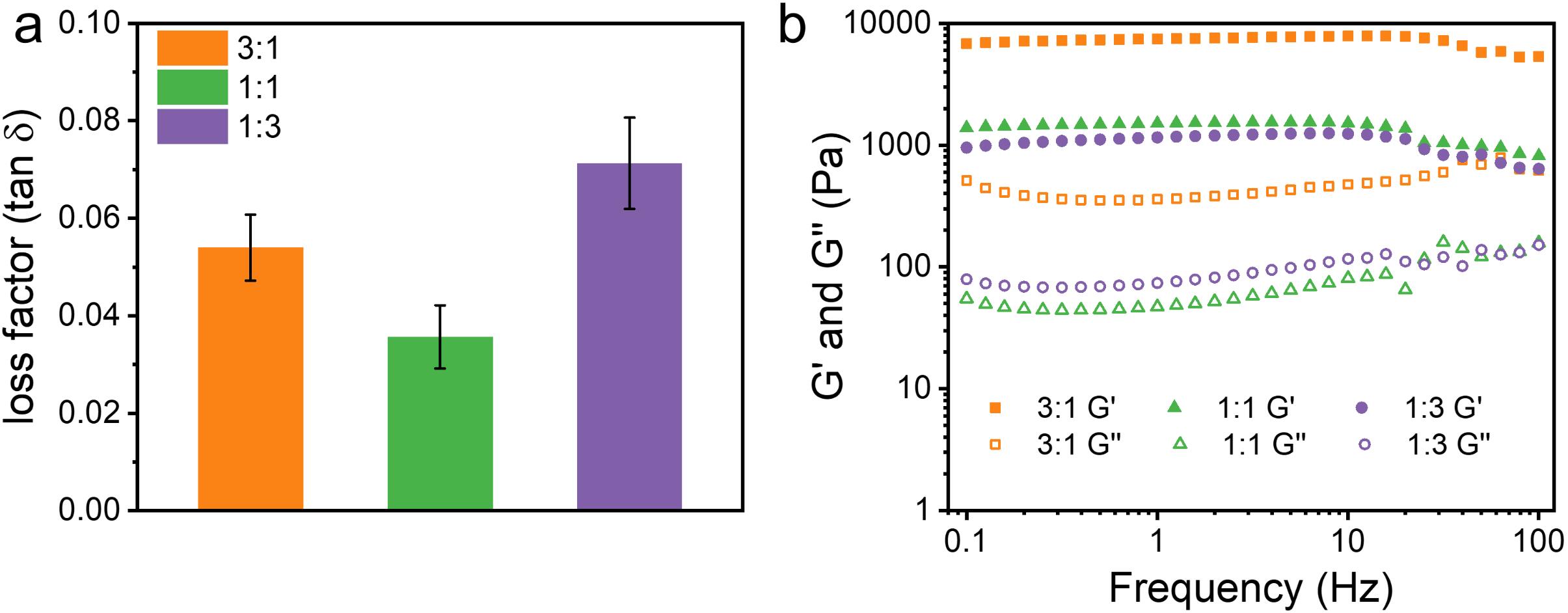


**Fig. S6** (**a**) Loss factor (tanδ) and (**b**) frequency dependence of MCG with different weight ratios of magnetic MMT to GNPs.


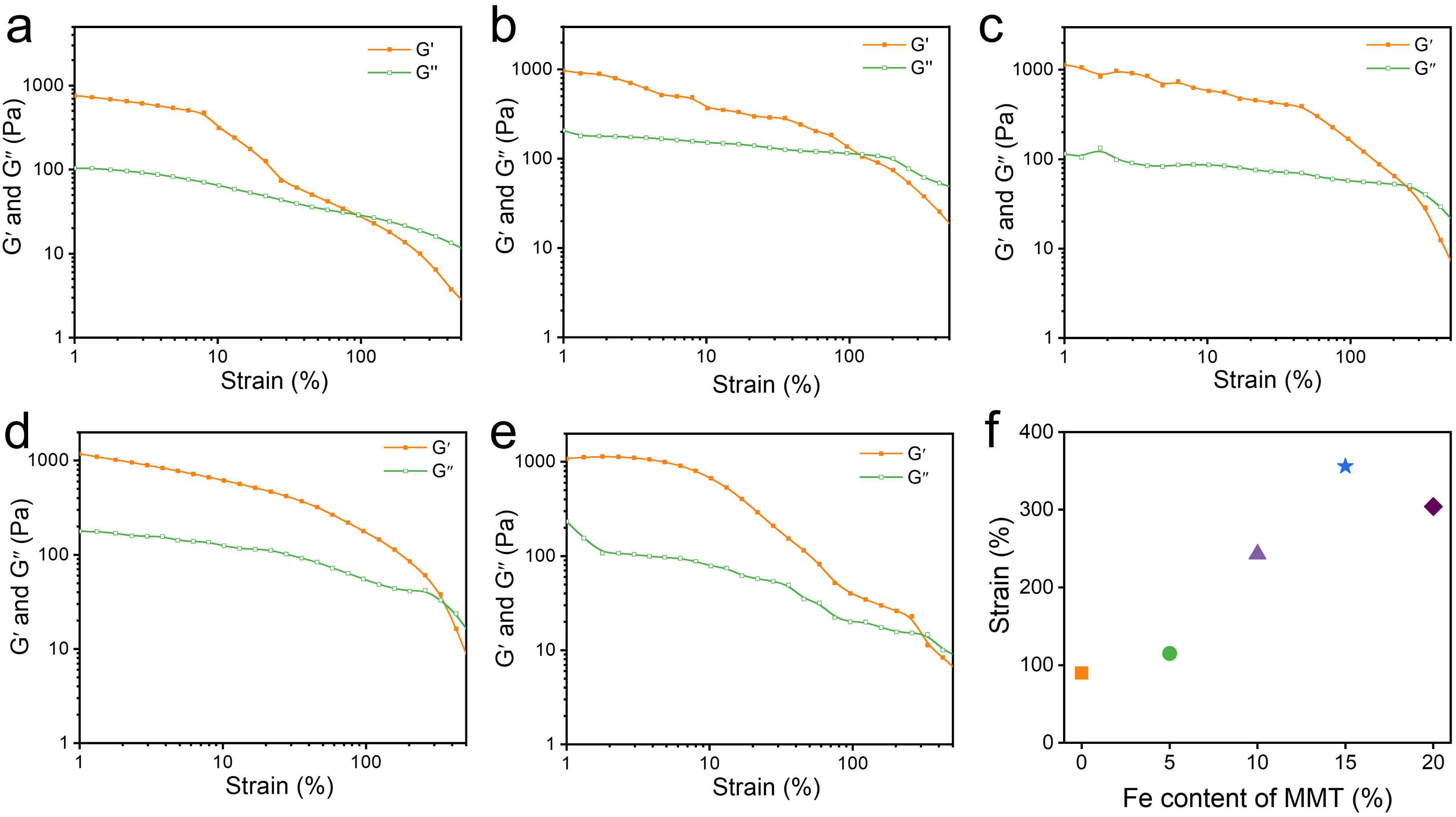


**Fig. S7** The G′ and G′′ on strain amplitude sweep for MCGs with different mass fraction of Fe (**a**) 0 %, (**b**) 5 %, (**c**) 10 %, (**d**) 15 %, and (**e**) 20 %. (**f**) The critical strain value of MCG with different mass fractions of Fe content. When the mass fraction of Fe content was 15%, the MCG showed the higher strain.


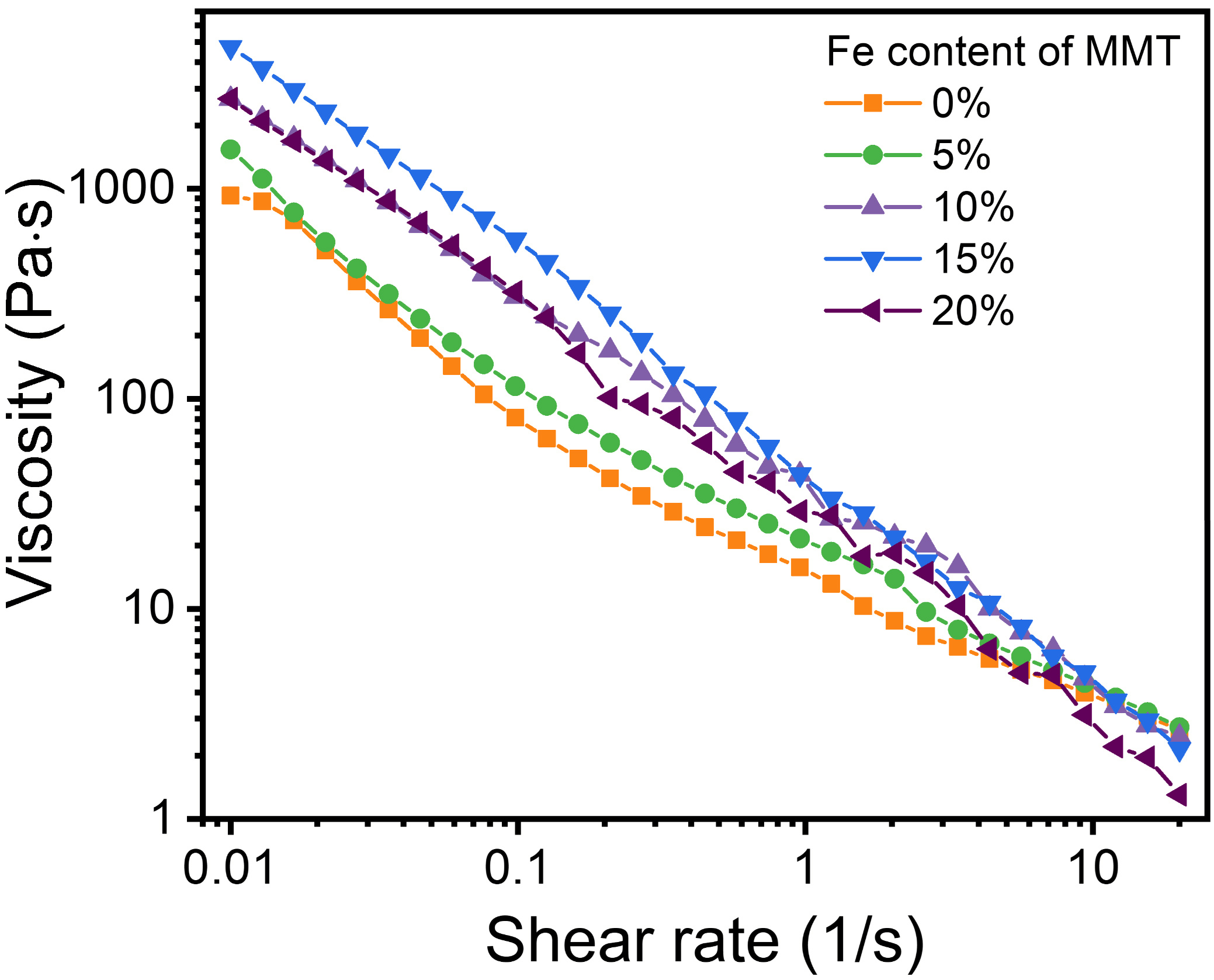


**Fig. S8** Shear-thinning behaviors of MMTs with different mass fractions of Fe.


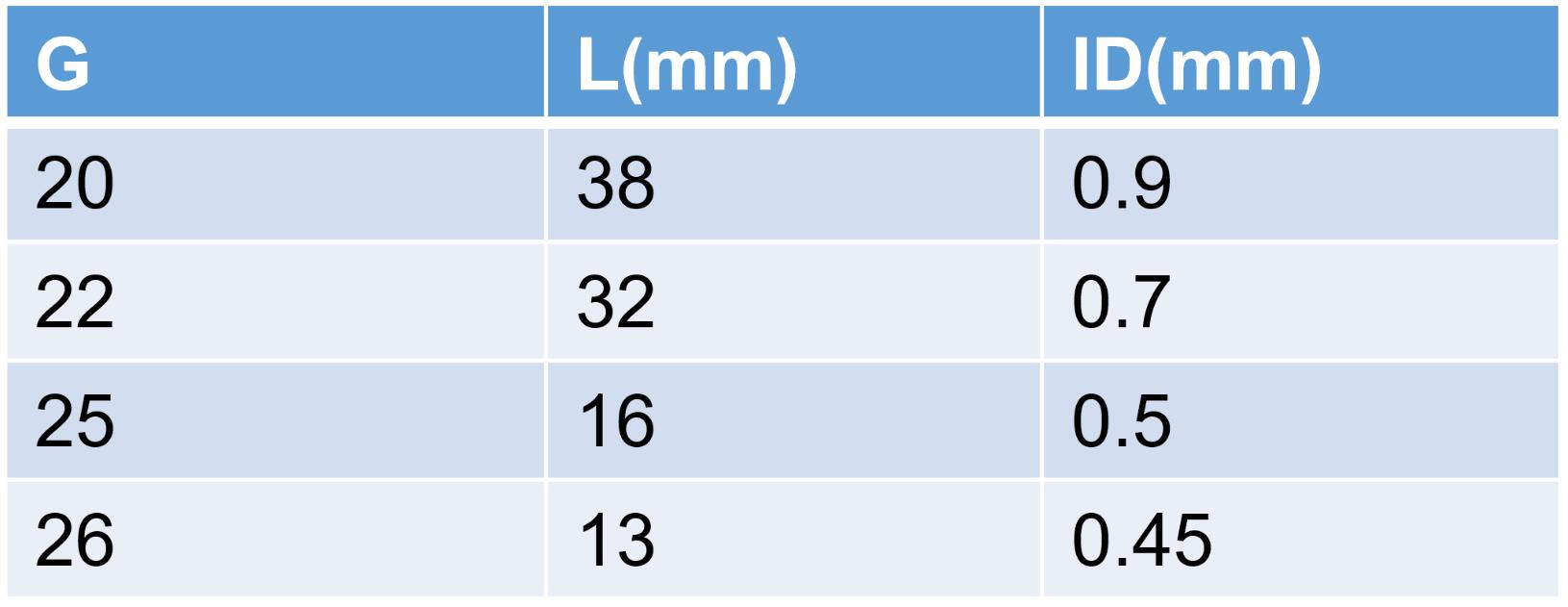


**Fig. S9** Table of needle dimensions.


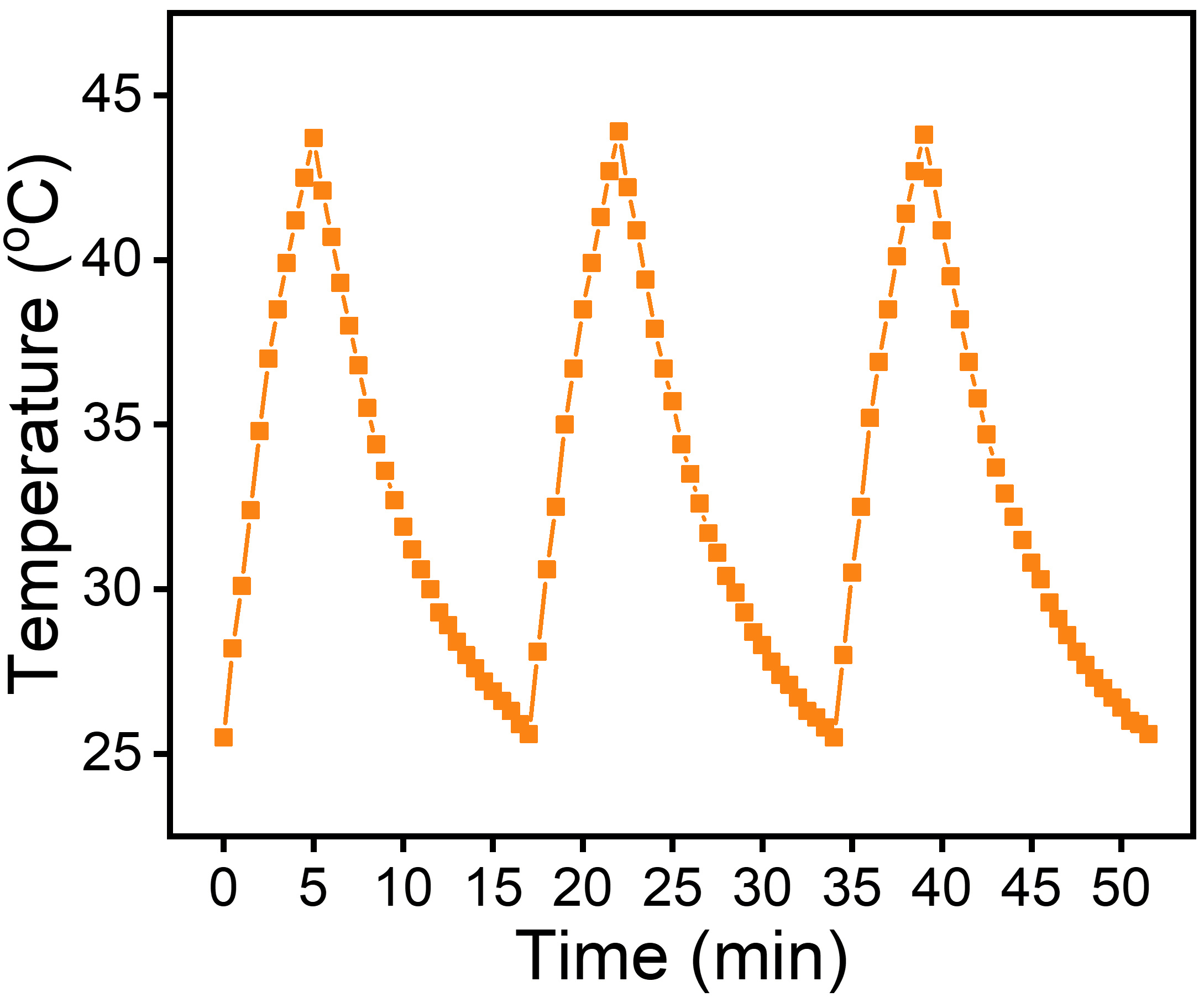


**Fig. S10** The magnetic hyperthermia conversion cycling test of MCG.


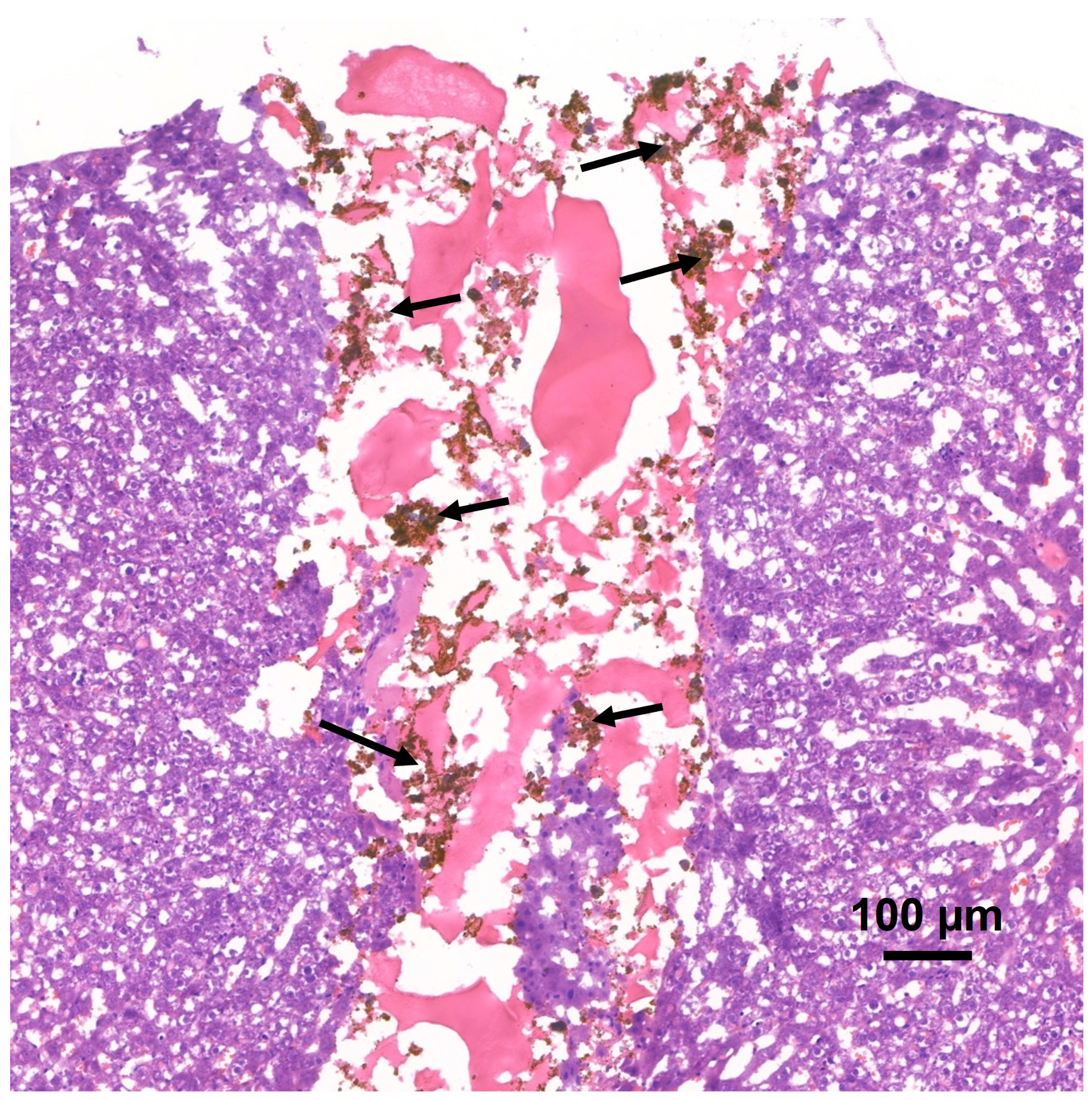


**Fig. S11** H&E images of damaged liver section. The remaining MCG were trapped in the wound gap, black arrows point to remaining MCG.


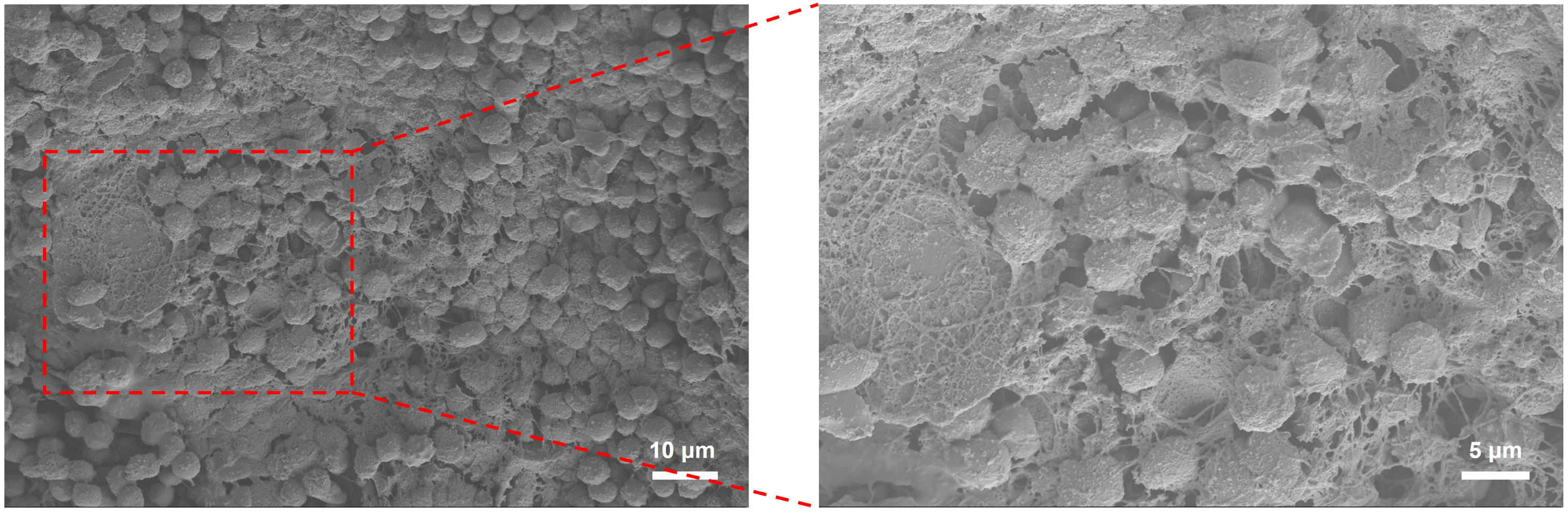


**Fig. S12** The SEM of that MCG leads to the formation of fibrin network.


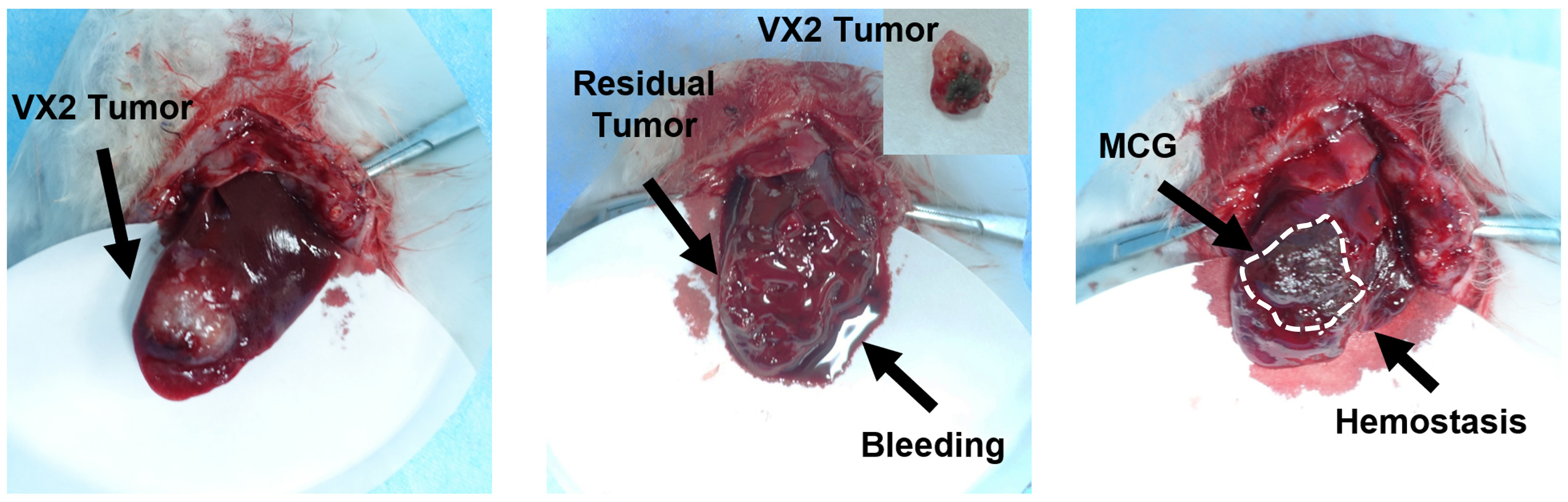


**Fig. S13** Photographs of hemostasis after hepatocellular carcinoma resection on the rabbit.


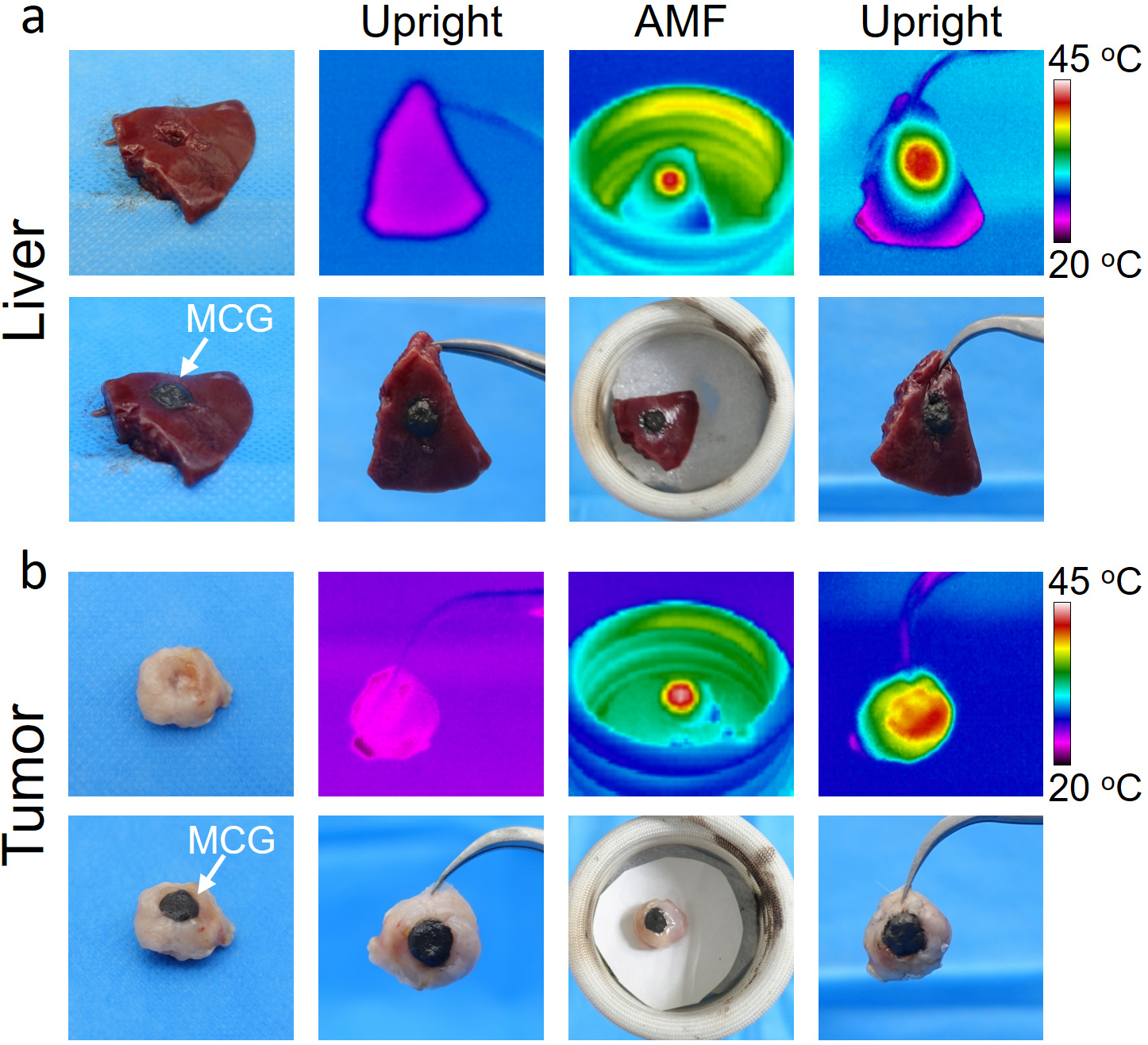


**Fig. S14** MCGs were attached to liver (**a**) or tumor (**b**) defects before and after applying AMF.


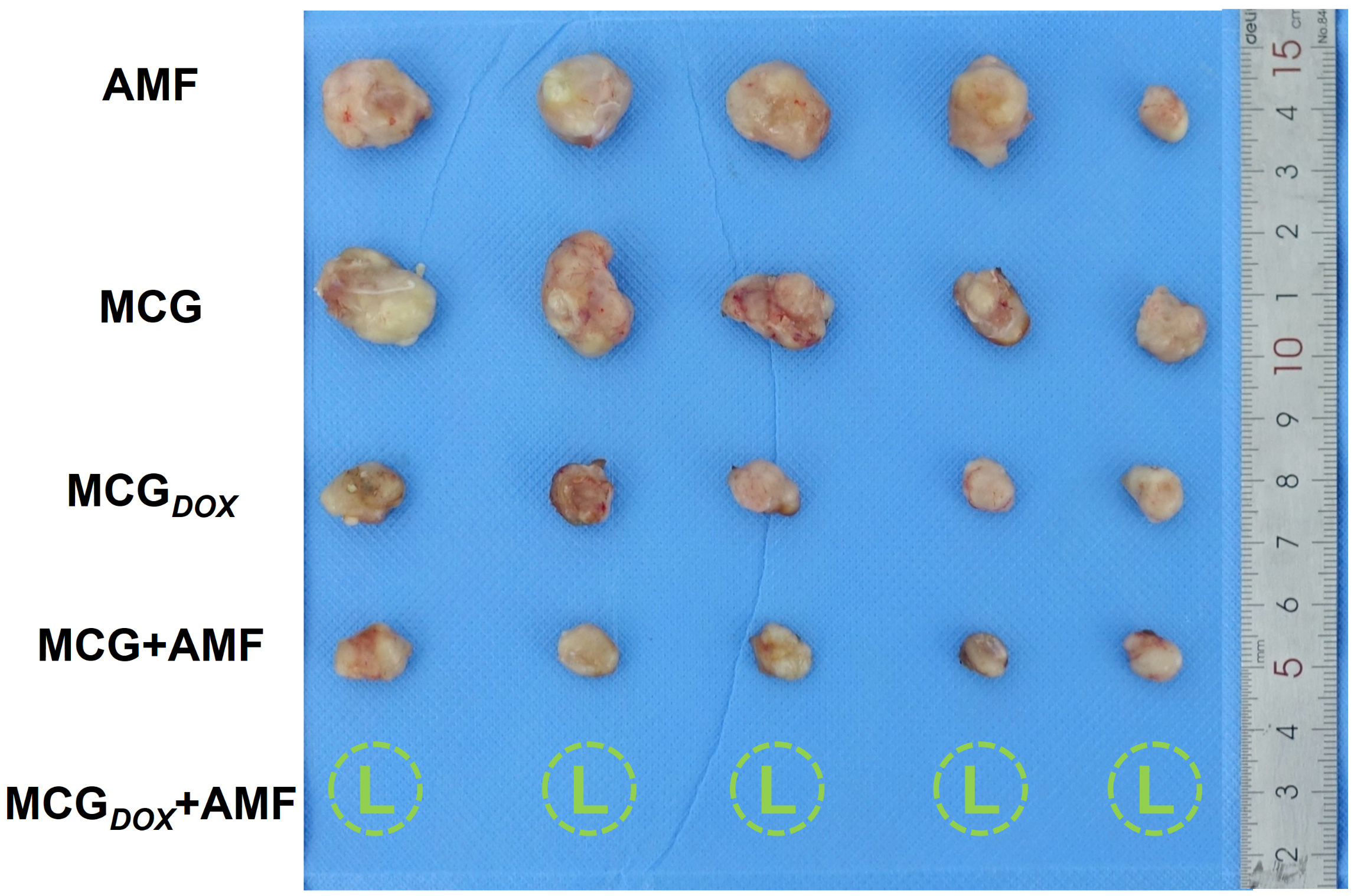


**Fig. S15** The digital images of excised HepG2 tumor after treatment at 14^th^ day, and the letter L existed inside of the dashed line circle represented live mice without tumor.


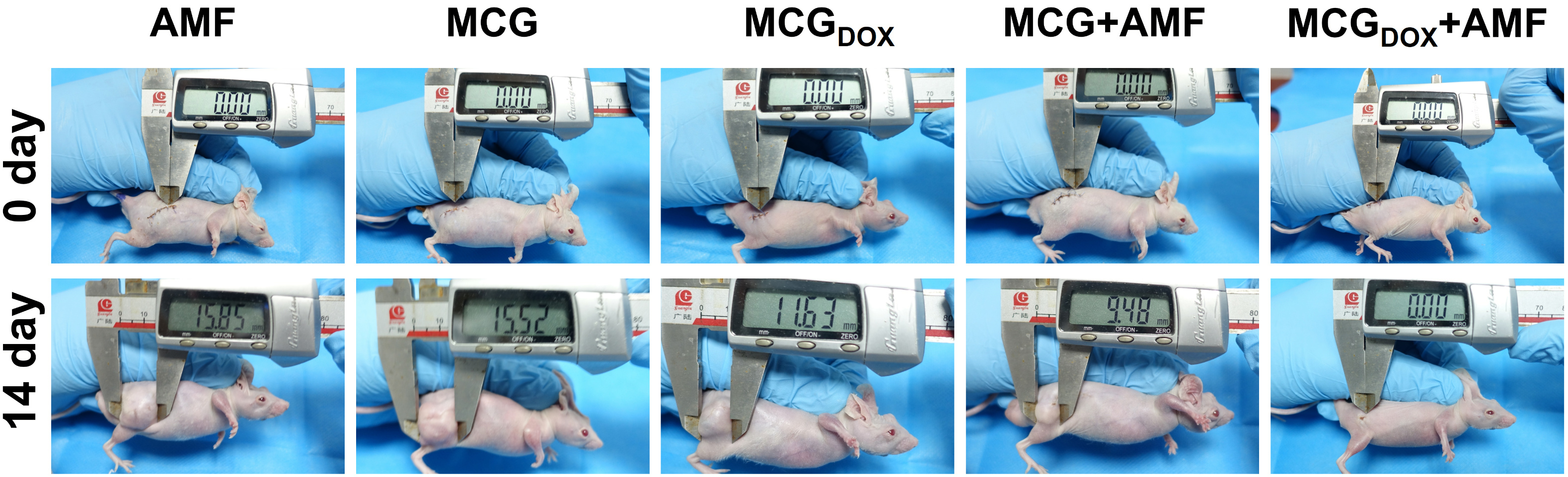


**Fig. S16** Photographs of mice from each group at 0 day and 14^th^ day.


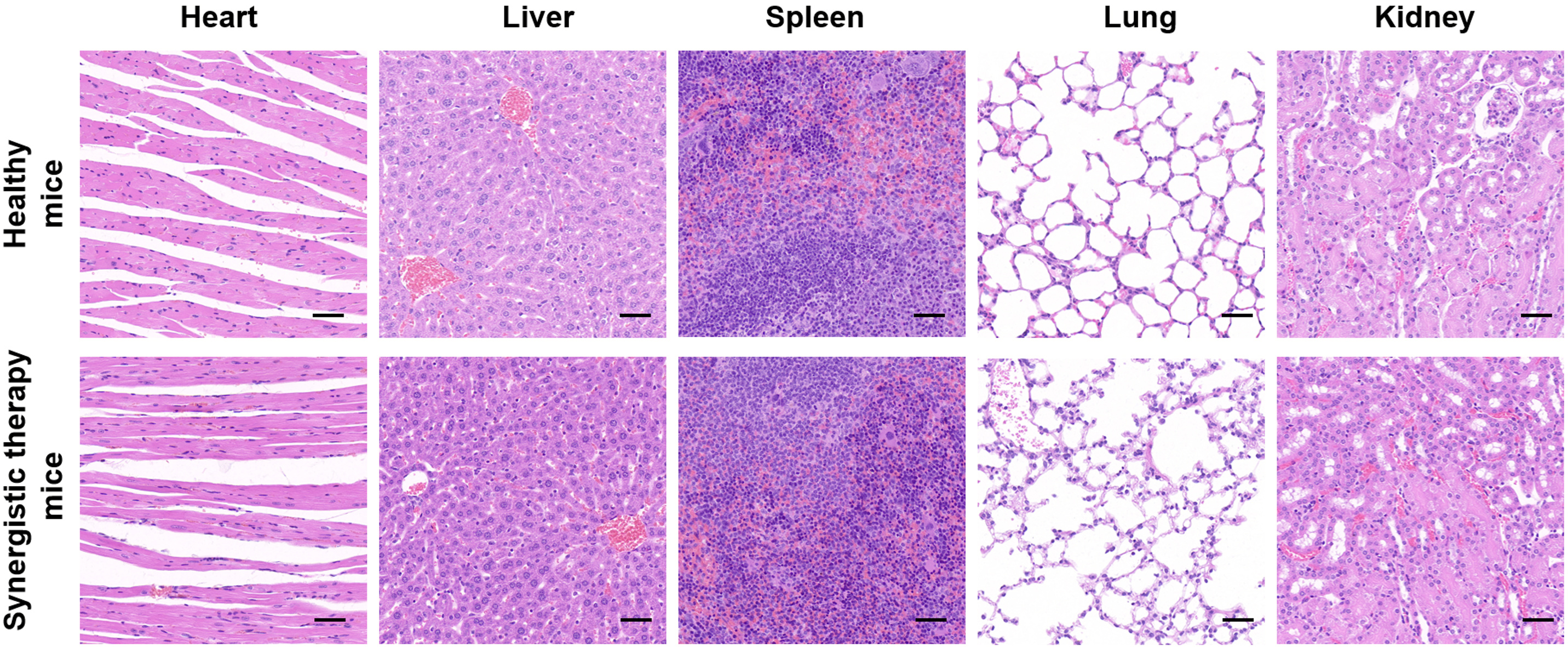


**Fig. S17** Representative H&E images of major organs (heart, liver, spleen, lung and kidney) of mice from the healthy group and MCG*_DOX_*+AMF group (Scale bar: 50 μm).
